# The Hippo effector TAZ promotes cancer stemness by transcriptional activation of SOX2 in head neck squamous cell carcinoma

**DOI:** 10.1038/s41419-019-1838-0

**Published:** 2019-08-09

**Authors:** Jin Li, Zhongwu Li, Yaping Wu, Yanling Wang, Dongmiao Wang, Wei Zhang, Hua Yuan, Jinhai Ye, Xiaomeng Song, Jianrong Yang, Hongbing Jiang, Jie Cheng

**Affiliations:** 1 Jiangsu Key Laboratory of Oral Disease, Nanjing Medical University, Nanjing, 210029 PR China; 2 Department of Oral and Maxillofacial Surgery, Affiliated Stomatological Hospital, Nanjing Medical University, Nanjing, 210029 PR China; 3 Department of Oral Pathology, Affiliated Stomatological Hospital, Nanjing Medical University, Nanjing, 210029 PR China

**Keywords:** Head and neck cancer, Cancer stem cells

## Abstract

The Hippo-TAZ signaling has emerged as a fundamental regulator underlying cancer stem cells (CSCs) stemness which intricately associates with local recurrence and metastatic spreading in head neck squamous cell carcinoma (HNSCC). However, the precise downstream targets of TAZ responsible for HNSCC CSCs maintenance remain largely underexplored. Here, we identified Sex determining region Y box 2 (SOX2) as a putative downstream target of TAZ to promote CSCs maintenance and tumorigenicity in HNSCC. Both TAZ and SOX2 were significantly enriched in CSCs subpopulation (CD44^+^CD133^+^) isolated from Cal27 and Fadu cells via fluorescence-activated cell sorting. TAZ knockdown significantly reduced expression of SOX2 at both mRNA and protein levels, whereas its ectopic overexpression markedly increased its abundance in HNSCC cells. Moreover, reintroduction of ectopic SOX2 abolished, at least in part, the reduced tumorsphere formation and tumorigenicity in vivo induced by TAZ knockdown. Mechanistically, transcriptional complex formed by TAZ and TEAD4 was recruited to two binding sites in SOX2 promoter, which in turn facilitated transcription of SOX2 in HNSCC cells. In addition, the abundance of TAZ and SOX2 was positively correlated in HNSCC clinical samples, and both upregulations of TAZ and SOX2 associated with the worst survival. Taken together, our data reveal a previously unknown mechanistic linkage between TAZ and SOX2 and identify SOX2 as a direct downstream target of TAZ in modulating CSCs self-renewal and maintenance in HNSCC. These findings suggest that targeting TAZ-SOX2 axis might be a promising therapeutic strategy for HNSCC.

## Introduction

Head and neck cancer comprises a heterogeneous group of malignancies that arise in the mucosal surfaces of the upper aerodigestive tract including oral and nasal cavity, pharynx and larynx as well as paranasal sinus^1^. The overwhelming majority of these tumors are squamous cell carcinoma (SCC) and account for approximately 3.8% of all cancer cases and 3.6% of cancer-related deaths worldwide^2^. Several etiological factors like tobacco smoking, alcohol consumption, chewing of betel quid and human papillomavirus (HPV) infection have been identified for HNSCC. Current clinical management of HNSCC including ablative surgery, radiotherapy and chemotherapy has yielded remarkable progress in the past decades. However, the long-term survival rate remains dismal and only 40–50% of patients survive for more than 5 years since initial diagnosis^3^. Molecular targeted therapies to treat advanced, recurrent or metastatic HNSCC are lacking or with limited success in selected patients^4^. These challenges largely hinge on our incomplete understanding about molecular tumorigenesis of HNSCC as well as its genetic and biological heterogeneities. Thus, in-depth investigations of molecular pathways underlying HNSCC pathogenesis will lead to novel effective therapeutics and optimal treatment guiding, ultimately improving patients survival and quality of life^5^.

Locoregional recurrence, cervical metastatic spread and chemoresistance largely account for therapeutic failure and cancer-related death in HNSCC^6^. These aggressive events have been revealed to be intricately associated with a unique cell subpopulation in bulk cancer, termed cancer stem cells (CSCs) or tumor-initiating cells (TICs) with potent self-renewal and tumor-seeding properties. These cells sustain tumor overgrowth and drive recurrence and metastatic dissemination^7,8^. Moreover, these unique characteristics have enabled CSCs as attractive and yet challenging therapeutic targets as evidenced by the facts that targeting key regulators or molecular pathways responsible for CSCs properties has yielded remarkable anti-cancer effects with promising translational potentials^9^. Previous pioneering work has documented several surface or functional markers for HNSCC CSCs including CD44, CD133, Bmi1, SOX2 and ALDH1^10–12^. Particularly, SOX2 (Sex determining region Y box 2), the key transcriptional factor preferentially expressed in embryonic and adult stem cells, has been demonstrated as an essential marker and regulator underlying these unique properties of CSCs from diverse cancer origins^13–15^. Previous reports have documented some essential clues to support SOX2 as a key CSC regulator in HNSCC^16–19^. However, the detailed mechanisms concerning how SOX2 itself is regulated in HNSCC remain incompletely known.

The Hippo signaling pathway has increasingly been recognized as a pivotal and indispensable mediator in tissue homeostasis, regeneration and tumorigenesis^20^. Dysregulation of Hippo signaling essentially contributes to cancer initiation, outgrowth, metastatic dissemination and therapeutic resistance. Upon Hippo inactivation, two downstream effectors transcriptional coactivator PDZ-binding motif (TAZ/WWTR1) and yes-associated protein (YAP) are translocated into nucleus whereby they drive transcription of target genes mainly by forming complexes with TEA domain DNA-binding family of transcription factors (TEADs)^21^. Importantly, we and others have reported that elevated TAZ promotes self-renewal and tumor-seeding potentials of CSCs and also confers CSCs-like properties on differentiated non-CSCs in diverse cancer contexts^22–24^. However, the accurate downstream targets responsible for TAZ in HNSCC CSCs self-renewal and maintenance remain underexplored yet.

Here, we sought to determine whether SOX2 was a novel downstream target of TAZ underlying CSCs properties in HNSCC. Our findings by integrating cellular experiments in vitro, tumor-forming assay in xenograft animal model as well as bioinformatics data mining provide evidence that TAZ enhances CSCs self-renewal and maintenance by direct transcriptional activation of SOX2 in HNSCC.

## Results

### SOX2 is a potential downstream target of TAZ in HNSCC

**Fig. 1:**
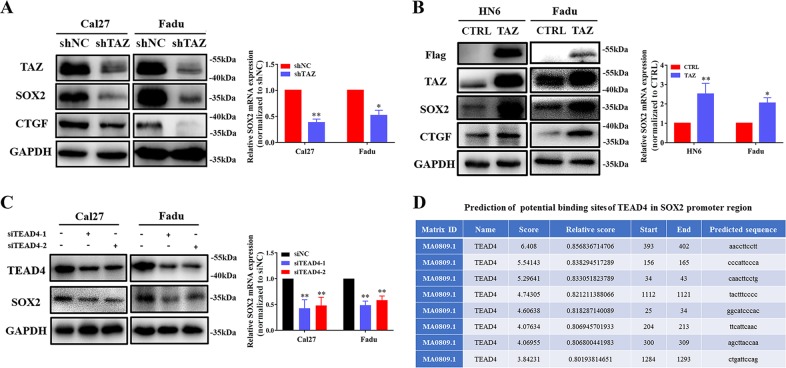
**TAZ regulates SOX2 expression in HNSCC cells**. **a** Reduced SOX2 expression upon shRNA-mediated TAZ knockdown was measured in both Cal27 and Fadu cells by western bot (left panel) and qRT-PCR (right panel) assays. **b** Enforced TAZ overexpression resulted in SOX2 upregulation in HN6 and Fadu cells. **c** Reduced SOX2 expression upon siRNA-mediated TEAD4 knockdown was measured in both Cal27 and Fadu by western bot (left panel) and qRT-PCR (right panel) assays. **d** Bioinformatics prediction of 8 potential binding sites of TEAD4 in human SOX2 promoter region via online JASPAR database (http://jaspar.genereg.net/). Data were presented as Mean ± SD from three independent experiments, *P < 0.05, **P < 0.01

Growing evidence has suggested that TAZ facilitates CSCs maintenance, self-renewal and expansion in multiple cancer contexts including HNSCC^22,23,25^. However, the accurate downstream targets responsible for TAZ underlying HNSCC CSCs properties remain largely underexplored. To address this, we set out to perform initial screen of dozens of putative CSCs regulators such as CD44, Nanog, SOX2, Bmi1 and ALDH1 as potential candidates via shRNA-mediated TAZ knockdown approach in vitro (data not shown). Among these candidates screened, SOX2 attracted our attentions due to the following reasons: its consistent and significant reduction upon TAZ silencing in both Cal27 and Fadu cells, its well-established roles in CSCs in squamous cell carcinoma as well as its intricate link with Hippo pathway in other biological contexts^11,15,17,26^. TAZ knockdown mediated by shRNA lentiviral constructs remarkably reduced both mRNA and protein abundance of SOX2, as well as CTGF (a well-known downstream target of TAZ) in vitro (Fig. 1a). In contrast, SOX2 was markedly upregulated in TAZ-overexpressing HNSCC cell lines (Fig. 1b). Complementarily, given TEADs as key transcriptional coactivators and mediators for TAZ functions and TEAD4 as a pivotal pro-oncogenic regulator in HNSCC^21,27^, TEAD4 knockdown via two independent siRNA oligonucleotides resulted in significant reduction of SOX2 expression in Cal27 and Fadu cells (Fig. 1c). Moreover, we employed online bioinformatics platform JASPAR (http://jaspar.genereg.net/) to predict potential binding sites of TEAD4 in human SOX2 promoter region (2000 bp upstream of TSS and 150 bp downstream of TSS) and identified eight potential binding sites (Fig. 1d), thus supporting the potential regulation of SOX2 by TEAD4. On the other side, two reports have revealed that SOX2 regulates YAP expression directly or indirectly by modulating Hippo signaling in diverse biological settings^26,28^. However, we introduced SOX2 into cells and failed to observe significant changes of TAZ protein and its phosphorylation upon SOX2 overexpression in 293 T, Fadu and HN6 cells (Fig. S1a–e). Moreover, the mRNA abundance of TAZ was not significantly affected by SOX2 overexpression as measured by qRT-PCR (Fig. S1b–f). Thus, our data suggested that TAZ might be not directly regulated by SOX2 in HNSCC. Collectively, these findings suggested that SOX2 might serve as a novel and important downstream target and mediator of TAZ during HNSCC tumorigenesis.

### SOX2 promotes CSCs stemness in HNSCC

**Fig. 2:**
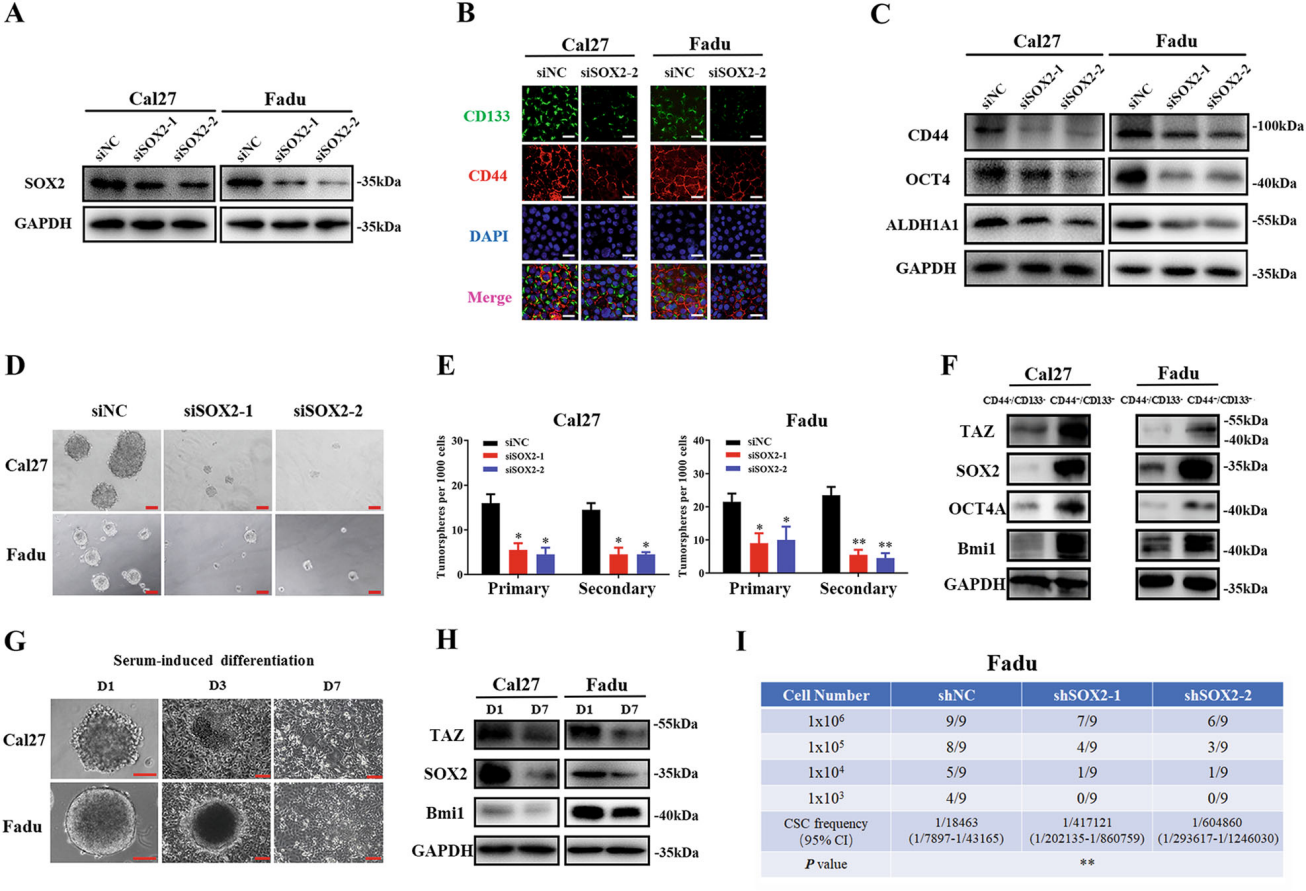
**SOX2 knockdown inhibits CSCs self-renewal and maintenance in HNSCC**. **a** Efficient SOX2 knockdown mediated by siRNA transfection in Cal27 and Fadu cells was confirmed by western blot assay. **b**, **c** Expression of multiple CSCs biomarkers including CD44, CD133, OCT4 and ALDH1A1 was measured by immunofluorescence staining (**b**, Scale bar: 50μm) and western blot (**c**). **d, e** Impaired tumorsphere formation was observed in cells transfected with two independent siRNAs targeting SOX2. Scale bar: 100 μm. Data were presented as Mean ± SD from three independent experiments, *P < 0.05, **P < 0.01, ANOVA analyses. **f** The protein abundance of TAZ and SOX2 was measured by western blot in both CSCs (CD44^+^CD133^+^) and non-CSCs (CD44^−^CD133^−^) subpopulations isolated from Cal27. **g** Tumorsphere was induced to differentiation into monolayer by serum from day 1 to 7. Representative images were shown. Scale bar: 100 μm. **h** The protein abundance of TAZ and SOX2 was measured by western blot in tumorsphere and serum-induced differentiated monolayer cells. **i** Tumor initiation was determined when cells transfected with SOX2 shRNAs were limitedly diluted and inoculated subcutaneously into NOD/SCID mice. Tumor was monitored and counted until 6 weeks after xenograft. Tumor-initiating frequencies were calculated via ELDA software (http://bioinf.wehi.edu.au/software/elda/). Data were presented as Mean ± SD from three independent experiments, *P < 0.05, **P < 0.01, ANOVA analyses

Given the essential roles of SOX2 in normal stem cells and malignant CSCs, we next sought to determine whether it was capable to modulate the properties of CSCs in HNSCC. We have previously identified a unique subpopulation with CSCs properties in HNSCC using surface markers CD44 and CD133 (CD44^+^CD133^+^ defined as CSCs) and demonstrated its robust self-renewal and tumor-initiating potentials both in vitro and in vivo^23^. As shown in Fig. S2, CD44^+^CD133^+^ CSCs subpopulation yielded much more and larger tumorsphere in vivo as compared to CD44^−^CD133^−^ subpopulation (non-CSCs). Then we utilized two independent siRNAs targeting human SOX2 to knockdown its expression and subsequently monitored the resulting phenotypic changes in vitro (Fig. 2a). In agreement with previous reports regarding SOX2 roles in cancer cell proliferation and apoptosis^18,29^, SOX2 knockdown by siRNA resulted in significant anti-proliferative and apoptosis-inducing effects in vitro as measured by CCK-8, BrdU incorporation and Annexin V-PI flow cytometric assays (Fig. S3). As shown in Fig. 2b, c, SOX2 depletion resulted in significant downregulation of several CSCs markers and mediators such as CD44, CD133 and ALDH1A1. Meanwhile, the potentials of tumorsphere formation were remarkably impaired after SOX2 depletion (Fig. 2d, e). In addition, following separation of CD44^+^CD133^+^ CSCs and CD44^−^CD133^−^ non-CSCs by FACS from Cal27 and Fadu cells, significant enrichments of TAZ, SOX2, OCT4 and Bmi1 protein expression were found in CD44^+^CD133^+^ CSCs as compared to CD44^−^CD133^−^ non-CSCs (Fig. 2f). Next, we conducted serum-induced in vitro differentiation of tumorsphere assay and determined the expression changes of TAZ and SOX2 during this process. As shown in Fig. 2g, h, when tumorsphere was cultured and then induced to differentiation with serum-containing media, these floating tumorsphere gradually attached the plates and grown into monolayers within 3 to 7 days. Accompanying this, SOX2 and TAZ protein expression were remarkably reduced in serum-induced differentiation cells (Day 7) as compared to tumorsphere (Day 1). More importantly, we utilized limited dilution and tumorigenic assay in vivo to further determine whether SOX2 was required for self-renewal and tumorigenesis in HNSCC. When stable SOX2-knockdown cells by shRNA lentiviral constructs were selected and transplanted subcutaneously with diverse amounts of cells, much lower incidence of tumor formation was observed in SOX2-depleted cells (Fig. 2i). Collectively, these findings together with others provide compelling evidence that SOX2 is critically involved in HNSCC initiation and progression at least in part by modulating CSCs stemness^17–19^.

### SOX2 is a key downstream pro-tumorigenic mediator of TAZ in HNSCC

**Fig. 3:**
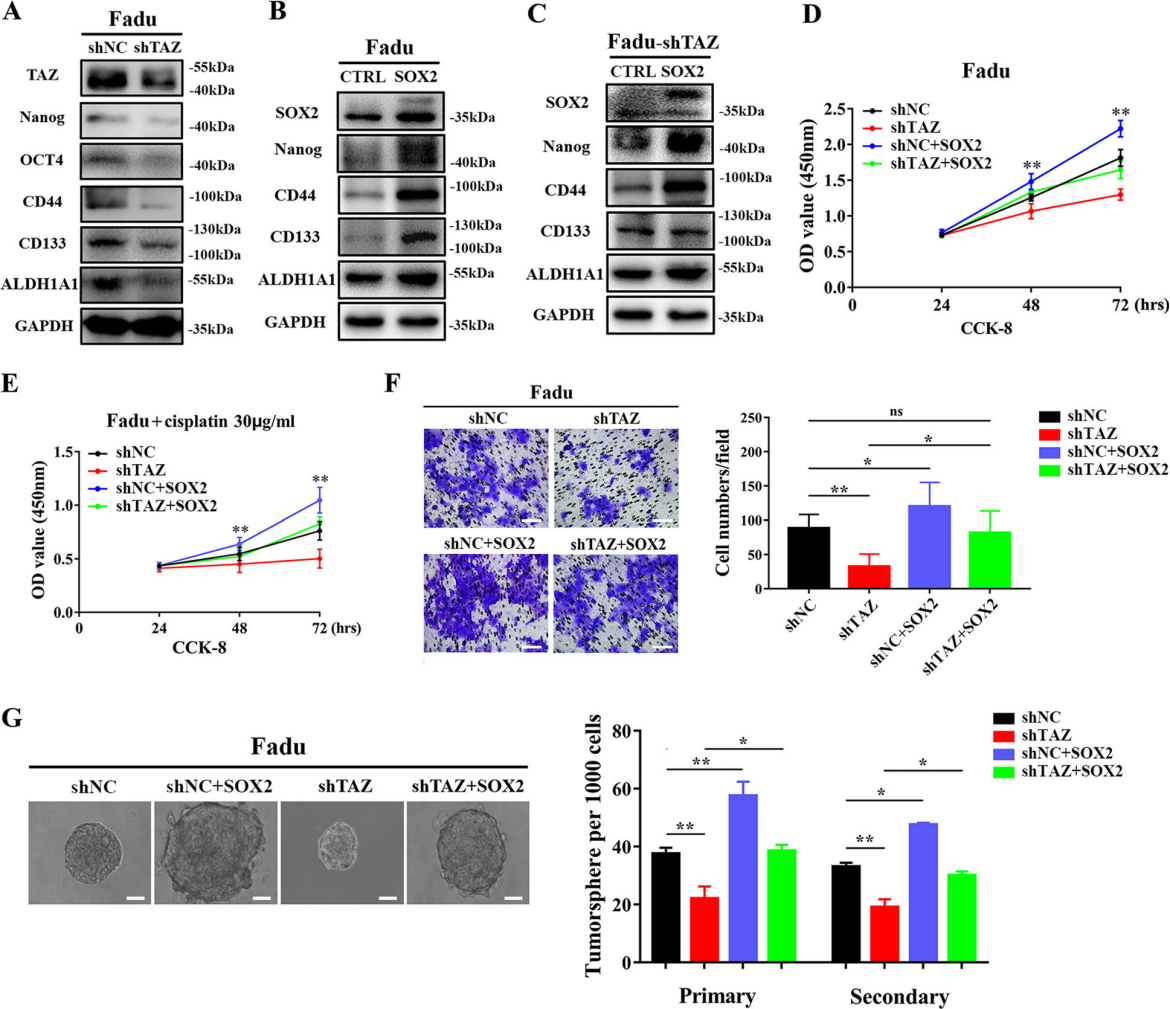
**Ectopic overexpression of SOX2 abrogates the effects induced by TAZ knockdown in HNSCC cells**. **a** The protein changes of multiple CSCs regulators and markers were measured in Fadu cells upon TAZ knockdown by western blot. Representative images were shown. **b** The protein changes of multiple CSCs regulators and markers were determined in stable SOX2 overexpressing Fadu cells by western blot. Representative images were shown. **c** The protein changes of multiple CSCs regulators and markers were determined in Fadu cells with stable TAZ knockdown following reintroduction of SOX2-overexpressing lentivirus by western blot. Representative images were shown. **d**, **e** Cell proliferation (**d**) and cisplatin (30 μg/ml, **e**) sensitivity in the indicated cells were measured by CCk-8 assay. **f** Cell invasion was measured by transwell chamber assay in indicated cells. Scale bar: 100 μm. **g** Tumorsphere formation was determined and compared in indicated cells. Scale bar: 100 μm

To further reinforce the notion that SOX2 is a novel downstream mediator of TAZ in HNSCC tumorigenesis, especially in the CSCs maintenance, we next performed rescue experiments and tumor-initiating assay via in vivo xenograft animal model. As shown in Fig. 3a, consistent with previous reports^23^, TAZ knockdown resulted in significant reduction of several CSCs markers like CD44, CD133 and ALDH1 in Fadu cells. Enforced overexpression of SOX2 by lentiviral constructs induced marked increase of these markers in Fadu cells (Fig. 3b). Notably, enforced overexpression of SOX2 largely rescued the expression of these CSCs markers in Fadu cells with stable TAZ knockdown (Fig. 3c). Additionally, as shown in Fig. 3d, e, our results from CCK-8 assays revealed that enforced overexpression of SOX2 largely abrogated the anti-proliferative effects as well as cisplatin sensitivity following TAZ depletion. Moreover, our data from transwell invasion and tumorsphere formation assays further indicated that reintroduction of SOX2 had the abilities to reverse, at least in part, the impaired invasiveness and self-renewal induced by TAZ depletion in vitro (Fig. 3f, g).

**Fig. 4:**
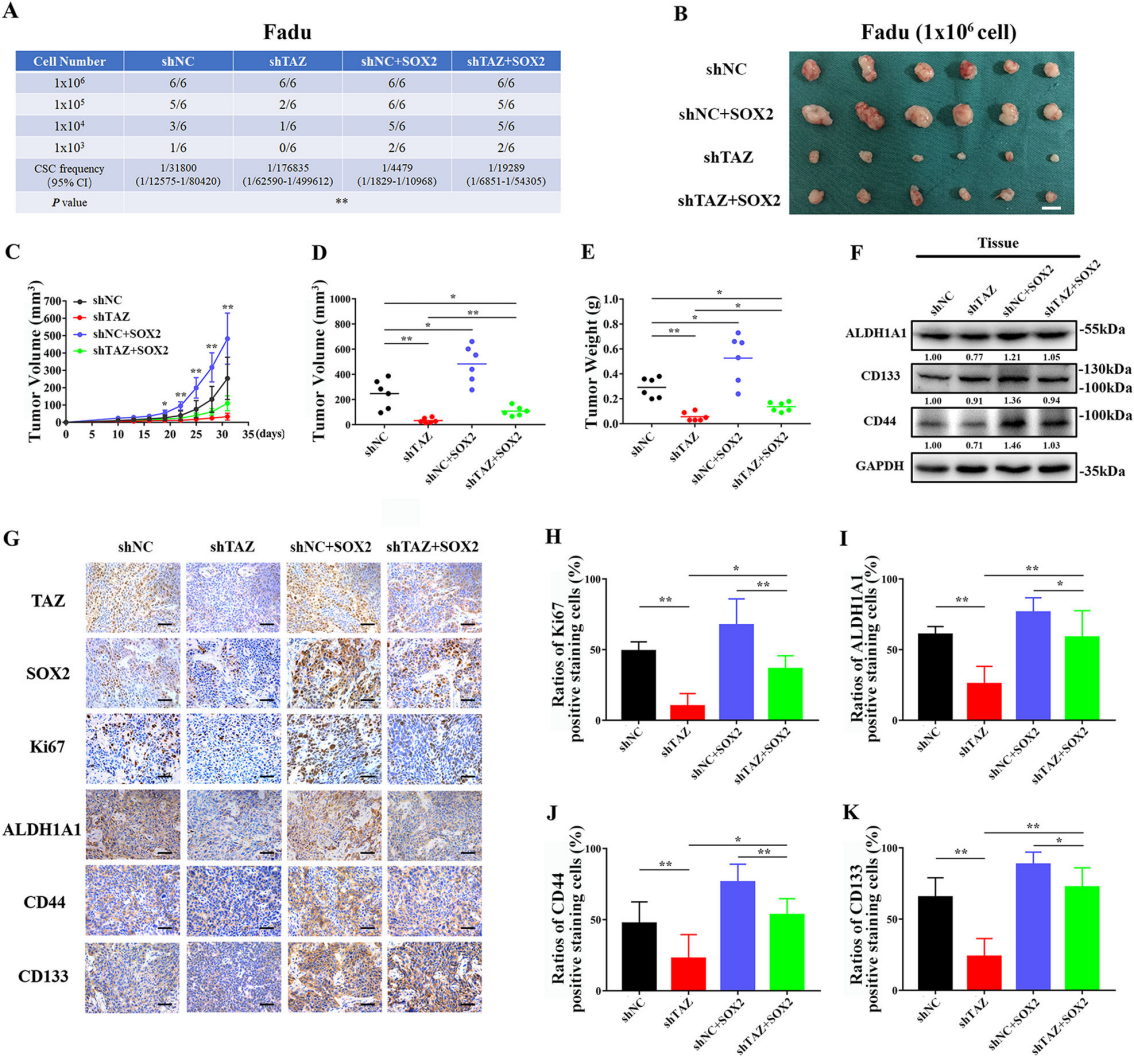
**Ectopic overexpression of SOX2 restores in vivo tumorigenicity in HNSCC cells with TAZ depletion**. **a** Tumor incidence was monitored in indicated cells as measured by limiting dilution tumorigenic assay. 10^3^–10^6^ cells were inoculated in the blanks of NOD/SCID mice. **b** Representative images of tumor xenograft samples derived from 10^6^ cells were shown. Scale bar: 1 cm. **c** Tumor volume was measured every three days after tumor initiation in animals inoculated with 10^6^ cells. **d**, **e** The final tumor volume (**d**) and weight (**e**) were measured in animals inoculated with 10^6^ cells. **f** Expression of CSCs markers like CD44, CD133 and ALDH1A1 was determined in samples derived from Fadu cells with TAZ or/and SOX2 manipulations. Representative images were shown. **g**–**k** Representative immunohistochemical staining images of TAZ, SOX2, Ki67, ALDH1A1, CD44, and CD133 in xenograft samples and their quantification data were shown. Scale bar: 100 μm

To recapitulate these in vitro findings in vivo, we utilized the limited dilution and tumorigenic assay. Various amounts of cells (10^3^–10^6^ cells) with stable TAZ knockdown, SOX2 overexpression and TAZ knockdown plus SOX2 overexpression were subcutaneously inoculated into both blanks of NOD/SCID mice. Consistently, as shown in Fig. 4a, TAZ depletion significantly impaired tumor-initiating potentials, while SOX2 overexpression enhanced tumor-initiating properties in vivo. Noticeably, when SOX2 was reintroduced into cells with stable TAZ knockdown, this manipulation was capable to partially restore their tumor-forming properties in vivo. As displayed in Fig. 4b–e, these samples were formed from transplantation with 10^6^ cells within 4 weeks. Data from volume during tumor growth, final volume and weight of tumor samples indicated that TAZ and SOX2 facilitated tumor overgrowth in vivo. Notably, enforced SOX2 overexpression was capable to partially abrogate the anti-growth effects resulted from TAZ knockdown. Then, tumor samples derived from Fadu cells with TAZ or/and SOX2 manipulations were subjected to further analyses. As shown in Fig. 4f, the expression levels of ALDH1A1, CD44 and CD133 were restored in samples derived from TAZ knockdown plus SOX2-overexpressing cells as compared to those from TAZ knockdown cells. Concomitantly, immunohistochemical staining coupled with quantitative analyses further supported that SOX2 overexpression had capacities to restore the expression abundance of these CSCs markers in HNSCC (Fig. 4g–k). Taken together, our findings strongly support that SOX2 is an important downstream mediator of TAZ in regulating CSCs self-renewal and tumorigenic potentials in HNSCC.

### TAZ-TEAD4 complex directly activates SOX2 transcription in HNSCC

**Fig. 5:**
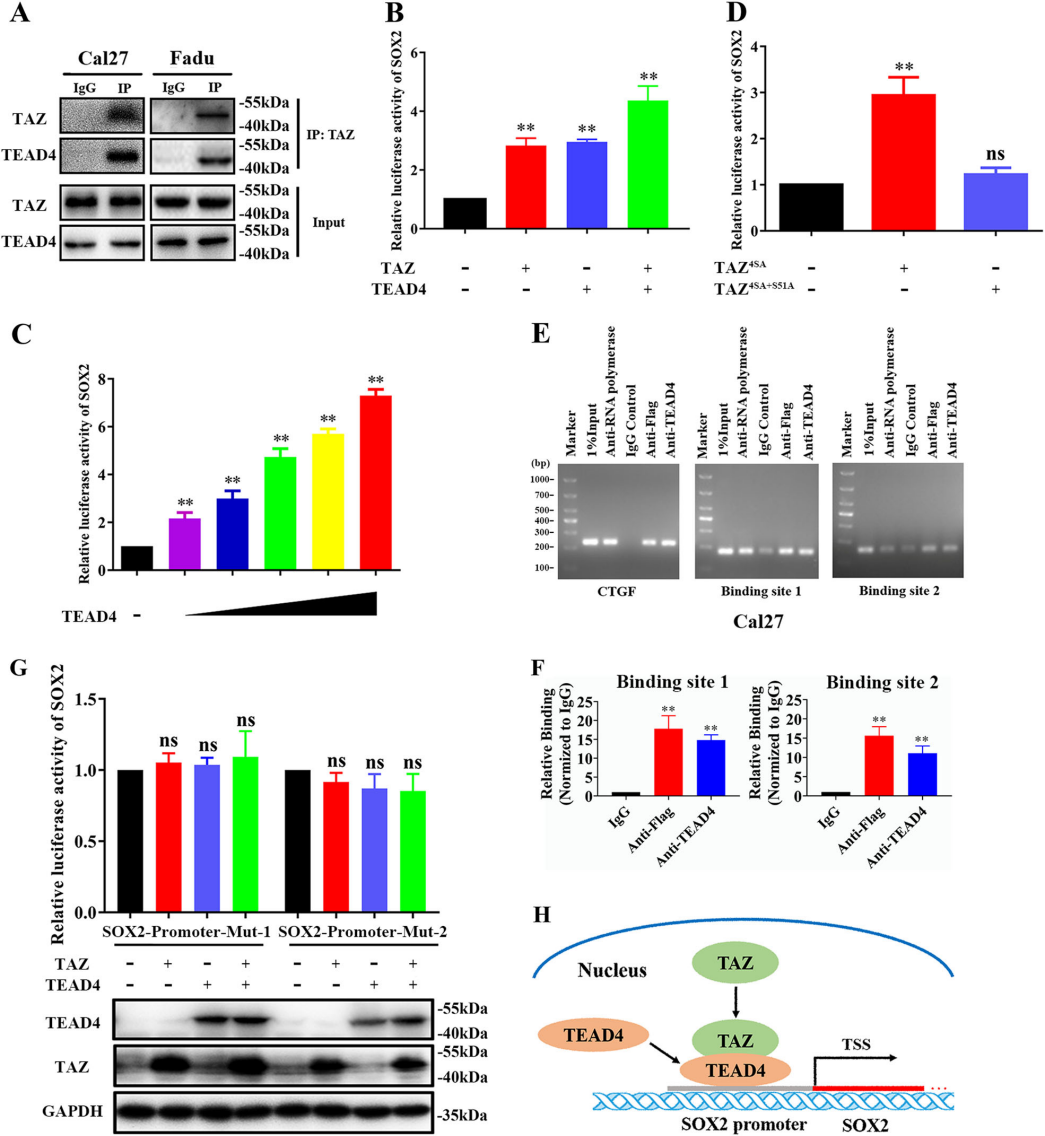
**TAZ activates SOX2 transcription by directly binding its promoter in HNSCC cells**. **a** Interaction between endogenous TAZ and TEAD4 protein was identified in Cal27 and Fadu cells immunoprecipitation (IP) assay using indicated antibodies. Representative images were shown. **b** Increased luciferase activities of SOX2 promoter reporter were detected in HEK293T cells transfected with TAZ, TEAD4 plasmid alone or in combination. **c** Increased luciferase activities of SOX2 promoter reporter were observed in HEK293T cells transfected with increased amount of TEAD4 plasmid. **d** TAZ constructively active plasmid (TAZ^4SA^) induced luciferase activities of SOX2 promoter reporter, while its mutant (TAZ^4SA+S51A^) failed. **e**, **f** Two binding sites in SOX2 promoter region for TAZ/TEAD4 in Cal27 were identified by ChIP PCR assay Cal27 cells transfected with TAZ were lysed and subjected to ChIP assay using indicated antibodies and PCR or qPCR primers **e**. The precipitated DNA samples were amplified by qPCR, and then compared **f**. 1% of total chromatin extract (input) was used as positive control, while normal IgG was used as negative control for IP assay. **g** Mutations of binding sites in SOX2 promoters significantly abrogated the increased luciferase activities induced by TAZ and TEAD4 plasmids. **h** Schematic model depicting that TAZ-TEAD4 complex binds with SOX2 promoter and in turn facilitates its transcription. Data were presented as mean ± SD from 3 independent experiments. *P < 0.05, **P < 0.01

Previous reports have demonstrated that Hippo effectors TAZ and YAP primarily bind with TEADs and act in concert to dictate downstream transcriptional outputs during diverse biological and pathological processes^20,21^. Here, we selected TEAD4 as the TAZ primary binding partner from four TEAD members due to its significant and consistent upregulation and key roles in HNSCC as we reported before^27^. Thus, we performed protein immunoprecipitation (IP) experiments in Cal27 and Fadu cells and found endogenous TEAD4 in complex immunoprecipitated by TAZ specific antibody (Fig. 5a). Next, we cloned the sequence of human SOX2 promoter by PCR and generated wide-type luciferase reporter of SOX2 promoter (WT, SOX2-luc) and conducted luciferase reporter assays. This reporter vector was simultaneously transfected with TEAD4-overexpressing or/and TAZ-overexpressing constructs into HEK293T. Significantly increased luciferase activities were observed in cells co-transfected with TEAD4 or TAZ overexpressing plasmids. As expected, much more luciferase activities were detected in cells transfected with both TAZ and TEAD4 vectors (Fig. 5b). Additionally, transfection with increased amount of TEAD4 constructs significantly enhanced luciferase activities in a dose-dependent manner (Fig. 5c). Moreover, TAZ mutant construct (TAZ^4SA+S51A^) without TEAD4 binding ability failed to increase luciferase activities of SOX2 promoter (Fig. 5d). Furthermore, we designed individual primers spanning 8 potential binding sites in SOX2 promoter (as shown in Fig. 1d), performed ChIP-PCR assay and found significant enrichments of TEAD4 binding in two sites (−309 to −300, −1292 to −1285, Fig. 5e). Consistently, we also designed specific primers, performed ChIP-qPCR assay and revealed that both TAZ and TEAD4 were significantly enriched in these two binding sites in SOX2 promoter (Fig. 5f). Furthermore, we generated two mutant luciferase reporters (SOX2-mut-1/2) by site-directed mutagenesis in these two sites in SOX2-luc WT plasmid. As shown in Fig. 5g, transfections of TAZ, TEAD4 overexpressing plasmid alone or in combination failed to induce significant effects on luciferase activities in cells co-transfected with SOX2 mutant promoter reporters. Taken together, as schematic illustration in Fig. 5h, our results indicate that TAZ-TEAD4 complex directly binds with SOX2 promoter and in turn facilitates its transcription in HNSCC.

### Prognostic significance of TAZ and SOX2 co-expression in HNSCC

**Fig. 6:**
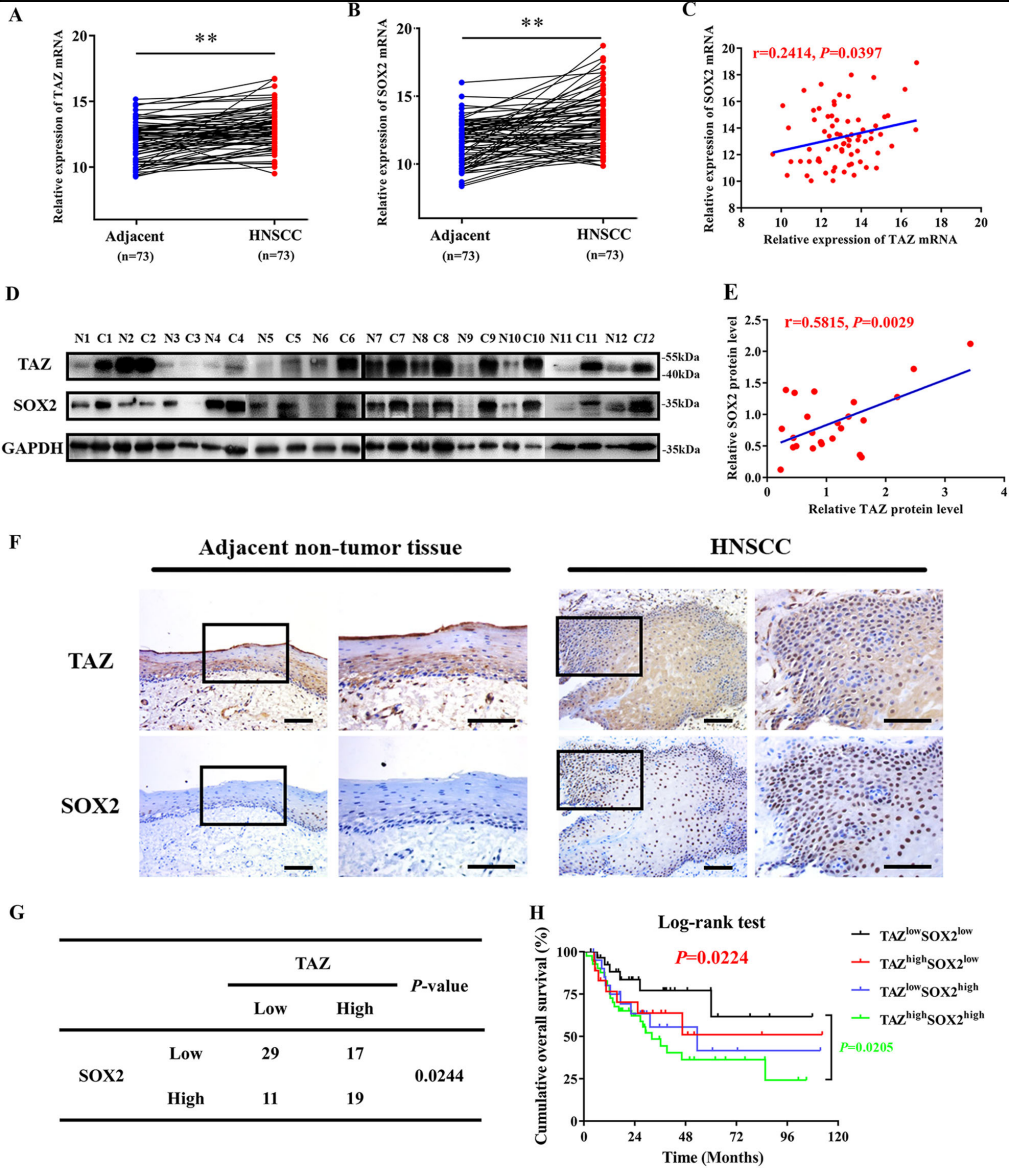
**Elevated TAZ and SOX2 associates with aggressive clinicopathological features and unfavorable patients’ prognosis in HNSCC**. **a**, **b** The mRNA expression levels of TAZ mRNA **a** and SOX2 mRNA **b** were measured in 73 fresh primary HNSCC samples and paired adjacent non-tumor tissue via qRT-PCR assay. *P < 0.05, **P < 0.01, paired *t*-test. **c** The correlation between TAZ mRNA and SOX2 mRNA in 73 primary HNSCC samples was estimated by using Pearson’s correlation. **d**, **e** The expression levels of TAZ protein and SOX2 protein were measured in 24 fresh HNSCC samples and paired adjacent non-tumor tissue via western blot assay **d**. Their correlation was estimated by using Pearson’s correlation **e**. **f** Representative immunohistochemical staining of TAZ and SOX2 in primary HNSCC and adjacent non-tumor tissue was shown. Scale bar: 100 μm. **g** The association between TAZ and SOX2 abundance in 76 primary HNSCC samples was estimated via Chi-square test. **h** Overall survival analyses of patients stratified based on combinational TAZ and SOX2 abundance in HNSCC were estimated by Kaplan–Meier method and compared with log-rank test. *P < 0.05, **P < 0.01

**Fig. 7:**
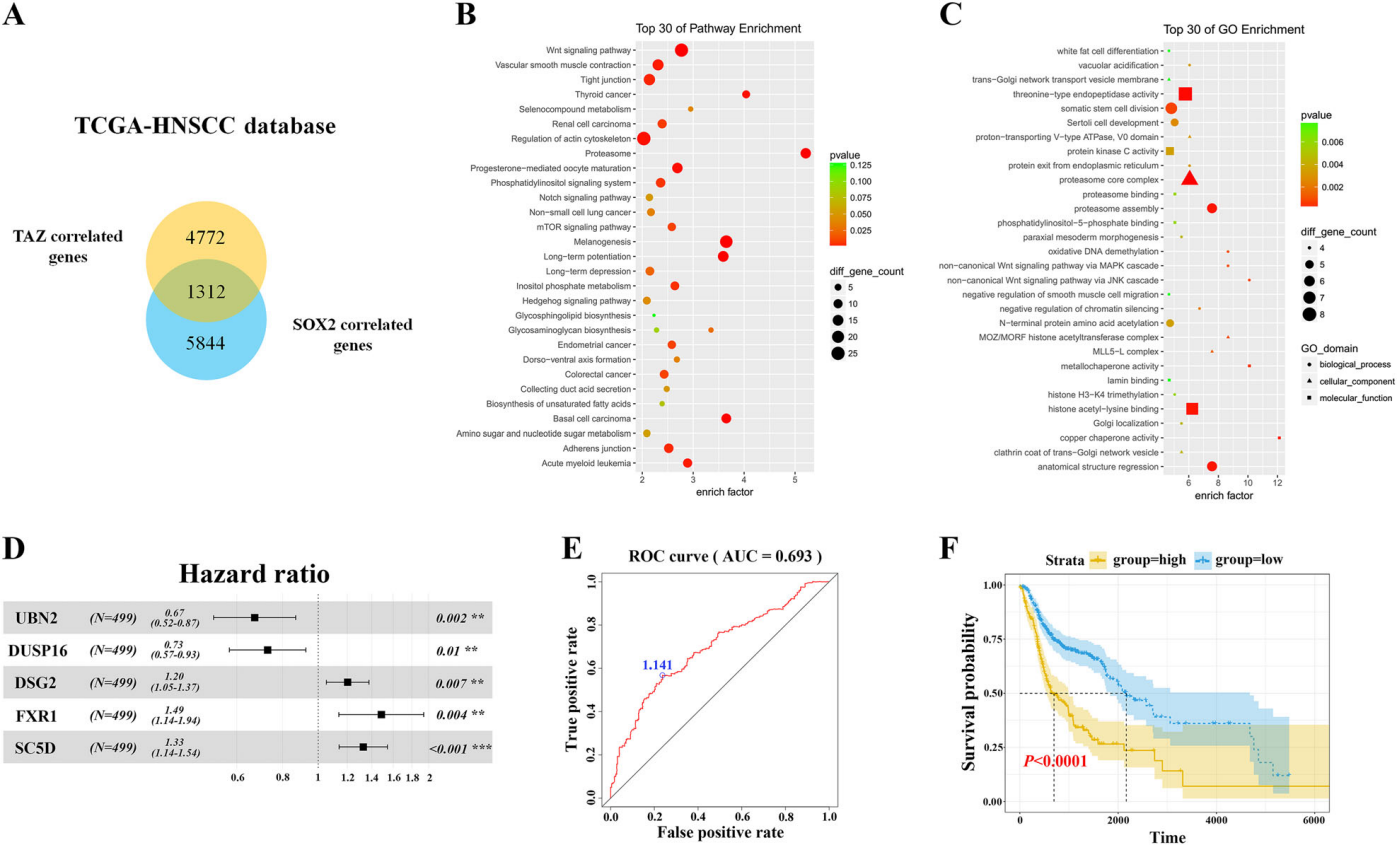
**TAZ and SOX2-correlated gene signature predicts survival of patients with HNSCC**. **a** A total number of 1312 overlapped genes were identified from TAZ-correlated and SOX2-correlated genes download in cBioPortal TCGA-HNSCC dataset. **b** These overlapped genes were significantly enriched in multiple CSCs-related pathways as assessed by KEGG analyses. **c** These overlapped genes were significantly enriched in CSCs-related pathways as assessed by GO analyses. **d** Detailed information regarding 5 candidate genes which were incorporated into a prognostic risk score. **e** The optimal cutoff value for the prognostic score was generated by ROC curve using TCGA-HNSCC as training cohort. **f** Kaplan–Meier plot indicated that patients with high scores had much inferior survival as compared to those with low scores (Log-rank test)

Having documented important roles of TAZ/TEAD4-SOX2 regulatory axis during HNSCC tumorigenesis, we next sought to determine whether their expression had prognostic significance in patients with primary HNSCC. The mRNA expression of TAZ and SOX2 in 73 primary HNSCC samples and paired adjacent non-tumor epithelial was measured by qRT-PCR assay. In addition, the protein levels of TAZ and SOX2 in 12 paired freshly collected HNSCC and adjacent non-tumor epithelial were also detected by western blot. As shown in Fig. 6a–d, significant upregulations of TAZ and SOX2 at both mRNA and protein levels were detected in HNSCC samples relative to their non-tumor counterparts. Moreover, positive correlations were found between TAZ and SOX2 at both mRNA and protein levels in these samples examined (Fig. 6c, e, Person correlation). Consistently, we mined the transcriptional profiling data of HNSCC samples from TCGA and GEO datasets and also found positive correlations between TAZ mRNA and SOX2 mRNA from three independent patient cohorts (Fig. S4). Next, we further determined the abundance of TAZ and SOX2 by immunohistochemical staining in archived HNSCC samples with detailed follow-up data available. As shown in Fig. 6f, obvious nuclear staining of TAZ and SOX2 was detected in cancerous cells while much less staining of SOX2 or cytoplasmic staining of TAZ was identified in non-tumor oral epithelial cells. According to our immunohistochemical scoring regime, as listed in Table S5, high TAZ expression was found in 36 patients while elevated SOX2 expression was detected in 30 patients. Significant associations were found between TAZ/SOX2 abundance and cervical nodal metastasis, as well as tumor size (Chi-square test, *P* < 0.05), while no significant correlations between TAZ/SOX2 and other clinicopathological parameters were identified. In addition, significant correlation between TAZ and SOX2 expression was found in these samples examined (Fig. 6g). Next, we applied Kaplan–Meier analyses to determine the prognostic significance of TAZ/SOX2 co-expression in HNSCC and revealed that patients with TAZ^high^SOX2^high^ expression had the worst prognosis as evidenced by the lowest overall survival among four subgroups categorized by TAZ and SOX2 expression (Fig. 6h). To further extend these findings, we exploited a bioinformatics approach by identification and filtering the overlapped candidates which were significantly correlated with TAZ or SOX2 in TCGA-HNSCC dataset. As shown in Fig. 7a, a total of 1312 overlapped genes were initially found between TAZ or SOX2- correlated genes in TCGA-HNSCC dataset. These genes were significantly enriched in diverse signaling pathways critically involved in CSCs self-renewal such as Wnt, Hedgehog and Notch pathways (Fig. 7b), and functional categories such as somatic stem cell division (Fig. 7c). Finally, to determine whether these TAZ/SOX2-correlated genes had prognostic significance in HNSCC, we developed a prognostic score comprising 5 genes (UBN2, DUSP16, DSG2, FXR1 and SC5D) by sequential univariate regression analysis, Robust likelihood-based modeling and multivariate regression analysis using TCGA-HNSCC dataset as training cohort (Fig. 7d). This risk score was calculated by the following formula: risk score = (−0.39864 × UBN2) + (−0.31743 × DUSP16) + (0.18477 × DSG2) + (0.39579 × FXR1) + (0.28306 × SC5D). The optimal cutoff for this score was 1.141 derived from the ROC curve using TCGA-HNSCC dataset (Fig. 7e). Then Kaplan–Meier analyses indicated that patients with high scores had markedly reduced survival as compared to those with low scores in TCGA-HNSCC dataset (*P* < 0.0001, Fig. 7f). Data from another two independent cohorts of HNSCC samples (GSE41613 and GSE42743) were further utilized as the testing and validation cohorts to verify the prognostic utility of this score. Consistent with the findings from the training set, patients with high scores had significantly lower OS ratios as compared to those with low scores in both testing and validation cohorts with favorable sensitivity and specificity (*P* = 0.00054, 0.00028; Log-rank test; Fig. S5). To reinforce the prognostic value of this score and rule out other confounding factors, we performed univariate and multivariate cox regression analyses and found that this score was an independent prognostic predictor for overall survival in the TCGA-HNSCC cohort (Table S5, Table S6). Taken together, our data indicate that TAZ/TEAD4-SOX2 axis associates with aggressive clinicopathological features and unfavorable patients’ prognosis in HNSCC.

## Discussion

Similar with other malignancies, HNSCC is characterized by a histologically heterogeneous population of cancer cells as evidenced that very small fraction of cells serves as CSCs or tumor-initiating cells that are critical for cancer initiation, recurrence, metastatic spreading and therapeutic resistance^9,10,12^. Dysfunctional Hippo signaling pathway has been increasingly recognized as a central mediator for tumorigenesis, as well as a potential therapeutic target in diverse cancer contexts^21^. In particular, TAZ has been identified as an essential modulator for CSCs self-renewal and maintenance in several human malignancies^22,23^. However, the precise downstream targets of TAZ and relevant underlying mechanisms for its function in HNSCC CSCs remain incomplete known. Here, our findings reveal that TAZ activates SOX2 transcription by directly binding to its promoter region and in turn facilitates CSCs maintenance and tumorigenicity in HNSCC.

Our previous results have showed that TAZ mediates self-renewal and maintenance of CSC in OSCC as evidenced by its enrichment in CSC subpopulation, impaired tumorsphere formation and reduced CSC percentage upon TAZ knockdown, as well as positive associations between TAZ expression and tumor aggressiveness in OSCC samples^23,30^. Moreover, enforced TAZ overexpression endowed non-CSCs with CSCs-related properties presumably by inducing epithelial-mesenchymal transition (EMT) in breast cancer and OSCC^22,23^. To extend these findings and bridge the gap between TAZ and CSCs stemness in HNSCC, we screened dozes of known CSCs regulators and found that SOX2 might be downstream target of TAZ in HNSCC as evidenced by its downregulation or upregulation followed by TAZ knockdown or overexpression in vitro. Consistent with previous findings regarding SOX2 in HNSCC stemness^16,18,19,31^, our data further confirmed the pivotal roles of SOX2 in CSCs maintenance in HNSCC as evidenced by impaired abilities of tumorsphere formation, reduced expression of CSCs markers and compromised tumor initiation in vivo following SOX2 knockdown. Additionally, both TAZ and SOX2 were significantly enriched in CSCs subpopulation and were significantly downregulated during serum-induced tumorsphere differentiation. More importantly, experimental findings derived from limiting dilution and tumorigenic assay in vivo further revealed the pivotal roles of TAZ and SOX2 required for HNSCC tumor initiation and overgrowth. Collectively, these findings provide compelling evidence to support the essential roles of TAZ and SOX2 in CSCs self-renewal and maintenance in HNSCC.

Previous studies have established that TAZ primarily functions as a transcriptional coactivator in complex with TEADs to mediate downstream transcriptional events^21,32^. Disruption of TAZ-TEADs binding blocked the major effects mediated by TAZ including oncogenic transformation, pro-proliferation and EMT in cancer^33^. Here, we identified TEAD4 as the key partner with TAZ to regulate SOX2 expression in HNSCC through IP assay, which was also consistent with its oncogenic functions and aberrant upregulation in cancer^27,34^. Subsequently, our results from luciferase reporter assays involving SOX2 promoter and its mutant as well as ChIP assays further confirmed the direct binding between TAZ/TEAD4 and SOX2 promoter in HNSCC. Noticeably, TAED binding sites in SOX2 promoter identified here were different from those found in other reports whereby two putative YAP/TEAD binding sites were found at upstream (−3759) and downstream ( + 5313) of SOX2 transcription start site (TSS) in murine osteosarcoma cells^35^. We reasoned that it’s conceivable that binding sites for transcriptional factors vary in diverse cell types and biological settings. More importantly, reintroduction of SOX2 into cells with TAZ stable knockdown abrogated, at least in part, the impaired proliferation, migration and tumorsphere formation, and in vivo tumor initiation and growth induced by TAZ depletion. Interestingly, several previous reports revealed that YAP1 transcriptionally activated SOX2 expression through a physical interaction with OCT4 to facilitate self-renewal of stem-like cells in lung cancer^28^. SOX2 antagonized Hippo pathway leading to exaggerated YAP functions to maintain stemness in osteosarcoma^15^. However, our data failed to support TAZ as a potential, direct downstream target of SOX2, which was consistent with previous report wherein TAZ was unaffected in SOX2-depleted osteoprogenitors^26^. Taken together, these in vitro and in vivo findings offer experimental support that TAZ-TEAD4 complex activates SOX2 transcription by direct binding with its promoter, which in turn modulates CSCs stemness in HNSCC. These findings identified another upstream regulator of SOX2 and added another layer of complexity to the transcriptional regulatory network governing CSCs unique characteristics.

The prognostic value of TAZ has been established in several human cancer including OSCC and HNSCC^23,25,30^. However, the expression pattern of SOX2 and its prognostic significance in HNSCC remain inconsistent and contradictory. Some reports found that SOX2 was frequently amplified in HNSCC and its upregulation associated with favorable survival, while others documented the opposite results^36–40^. In our patient cohort, we failed to identify positive or negative association between SOX2 expression and patient survival (data not shown). These discrepancies might be due to sample size, tumor heterogeneity as well as methods of patient stratification. Nonetheless, our results revealed that patients with TAZ^high^/SOX2^high^ had the worst survival ratios as compared to other patient subgroups. Of course, further studies are needed to resolve the inconsistency regarding the prognostic value of SOX2 in multiple, large and independent HNSCC cohorts. Noticeably, we developed a prognostic score based on TAZ/SOX2-correlated genes via bioinformatics and statistical approaches and found that this score had potent power to stratify HNSCC patients into subgroups with favorable or inferior survival. This suggests that the regulatory network modulated by TAZ and SOX2 in HNSCC might not only intricately contribute to tumorigenesis, but also might be exploited as a novel prognostic biomarker with translational promise in the clinic.

In conclusion, our data reveal a previously unknown mechanistic linkage between TAZ and SOX2 and identify SOX2 as a direct downstream target of TAZ in modulating CSCs self-renewal and maintenance in HNSCC. These findings suggest a novel therapeutic approach to target TAZ/TEAD4-SOX2 signaling axis in HNSCC.

## Materials and methods

### Cell culture and regents

A panel of HNSCC cell lines including Cal27, Fadu, HN6 and human embryonic kidney 293 T (HEK293T) was used here. Cal27, Fadu and HEK293T were obtained from American Type Culture Collection (ATCC, Manassas, VA, USA) and authenticated by short tandem repeat profiling at regular intervals. HN6 was a generous gift from Prof. Wantao Chen (Shanghai Jiaotong University). All cancerous cells lines were maintained in DMEM/F12 (Gibco) supplemented with 10% fetal bovine serum (Gibco) and 1% penicillin/streptomycin at 37 °C in 5% CO_2_. *Mycoplasma* detection was routinely performed during the whole course of this study. All regents were purchased from Sigma-Aldrich unless otherwise stated.

### Small interference or hairpin RNA, DNA constructs, viral production and transfection/infection

Two independent sequences of siRNA or shRNA targeting human SOX2 and TEAD4 mRNA (detailed sequences were listed in Table S1) were designed and synthesized from GenePharma company (Shanghai, China). These siRNAs were transiently transfected into cells with lipofectamine 2000 (Invitrogen) at final concentration of 100 nM unless otherwise specified. Two short hairpin RNAs (shRNAs) against human TAZ mRNA or TAZ overexpression lentiviral construct tagged with single N-Flag was generated as we previously reported^23^. The TAZ mutant plasmids (TAZ^4SA^ and TAZ^4SA+S51A^) were kindly gifted from Prof. Kunliang Guan^41^. The human full-length SOX2 or TEAD4 cDNA with 3 × Flag was subcloned into lentiviral plasmid pLenti CMV/Puro and then verified by direct sequencing. Lentiviral particles were prepared by transiently co-transfecting HEK293T cells with individual lentiviral constructs and controls together with packaging and envelope plasmids (pCMV-VSV-G and pCMV-Δ8.2) using the calcium-phosphate method. These viral supernatants were filtered, concentrated and stored until use. For transient transfection assay with siRNA or plasmids, cells were harvested at 48 h for further experiments. To gain stable clones after infections with shRNA or overexpression lentiviral vectors, cells were selected with puromycin (2–5 μg/ml, Sigma) for at least one week.

### RNA extraction, and quantitative real-time PCR (qRT-PCR)

Total RNA of tissue specimens or cells was extracted with Trizol reagent (Invitrogen) and then subjected to transcription into cDNA by PrimeScript™ RT Master Mix (Takara) according to the manufacturer’s instructions. PrimeScript^TM^ RT-PCR kit (Takara) was used for qRT-PCR reactions, as we described previously^23,42^. Endogenous 18 S RNA or GAPDH was used for data normalization. All qPCR primers used were listed in Table S2.

### Cell viability, proliferation and invasion assay

Cell proliferation and viability were assessed by absorbance using CCK-8 cell viability assay (Cell Counting Kit-8, Dojindo, Japan) and BrdU incorporation assay according to manufacturer’ instructions. BrdU^+^ cells were identified under fluorescent microscopy, photographed and counted via ImageJ software. Cell invasion was assessed using transwell chambers with 8-μm pore size (Corning) with pre-coated Matrigel (BD Pharmingen) as we described previously^43^.

### Flow cytometry and fluorescence active cell sorting (FACS)

Flow cytometry for cell apoptosis and fluorescence-activated cell sorting were similar as we reported previously^23^. Briefly, for apoptosis detection, cells were trypsinized, dissociated into single cell suspension, then assayed with Annexin V: PE Apoptosis Detection Kit (BD Bioscience) for flow cytometry. For FACS, single cell suspension was incubated with CD44 (560890, BD Pharmingen, 1:100) and CD133/1 (AC133, Miltenyi, 1:100) and two subpopulations of CD44^+^CD133^+^ and CD44^−^CD133^−^ was separated when corresponding immunoglobulins was used for blank control. All data were collected and analyzed by BD FACSuite software.

### Western blot and immunoprecipitation (IP)

Western blot analyses were routine performed as described previously^23^. GAPDH was used as a loading control. For co-IP assay, cells were lysed by the Western & IP Lysate Buffer (Beyotime, China) supplemented with 1% protease inhibitor (Roche) on ice for >15 min. Cell lysate was centrifuged under 4 °C for 10 min at 12,000 rpm, and the supernatant was incubated with primary antibodies and protein A/G agarose beads (Thermo Fisher Scientific) with rotating at 4 °C overnight. The pellet was washed at least three times with IP lysate buffer on ice and then subjected to western blotting analysis. All antibodies used were listed in Table S3.

### Luciferase reporter assay

The promoter sequence (2065 bp) upstream of the transcriptional start site of human SOX2 was subcloned into a luciferase reporter plasmid and verified with direct sequencing. Two putative binding sites between TEAD4 and SOX2 were individually mutated using QuikChange® Lightning Site-Directed Mutagenesis Kits (Stratagene) and verified by direct sequencing. The 293 T cells were transiently transfected with pGL-SOX2, TAZ or TEAD4 plasmids and phRL-CMV plasmid (Promega) using lipofectamine 2000 (Invitrogen). Cells were harvested 24 h after transfection and assayed for Firefly and Renilla luciferase activity using the Dual-Luciferase reporter system (Promega). Data were presented as the ratios of Firefly to Renilla luciferase activity.

### Chromatin immunoprecipitation (ChIP) assay

For ChIP assay, Cal27 cells that had been transfected with Flag-TAZ for 48 h were harvested and lysed using the EZ-ChIP^TM^ Chromatin Immunoprecipitation Kit (Millipore) according to the manufacturer’s instructions. Isolated and purified chromatin was incubated overnight with antibodies for TEAD4 (Abcam), Flag (Sigma), RNA polymerase (Millipore), or normal mouse IgG (Millipore). RNA polymerase was utilized as positive control and normal mouse IgG was used as negative control. The PCR or qPCR primers for detecting CTGF, SOX2 binding site 1 and 2 were listed in the Table S4.

### Tumorsphere formation assay

The disassociated single cells (10^4^/ml) were cultured in serum-free DMEM/F-12 supplemented with B27, 20 ng/ml EGF (BD Bioscience) and 10 ng/ml bFGF (BD Bioscience) and grown in ultra-low-attachment plates (Corning) at routine conditions for 7–10 days. For in vitro serial passages, these tumorsphere were harvested and further dissociated into single cells by 0.1% trypsin and gentle pipette, and then filtered, re-plated to form secondary sphere in aforementioned medium. The tumorsphere with diameter larger than 50 μm was counted.

### HNSCC xenograft animal model

All animal models involved in this experiment were in accordance with the institutional animal welfare guidelines and protocols were approved by Institutional Animal Care and Use Committee of Nanjing Medical University. Six-week-old female NOD/SCID mice were purchased from Model Animal Research Center of Nanjing Medical University and maintained in the specific pathologic-free animal facility. Cancer cells were trypsinized, counted and diluted with equal number (1 × 10^6^, 1 × 10^5^, 1 × 10^4^, 1 × 10^3^) in 200 μL media (100 μL PBS plus 100 μL PBS Matrigel) and then subcutaneously injected into both flanks of each animal (6 animals per experimental group). Tumor initiation and growth were monitored and recorded every three days. Tumor volume was calculated with the formula: volume = a × b^2^/2. Tumor weight was weighed and recorded after animals sacrificed. Tissue samples were fixed in formalin or maintained in liquid nitrogen for further analyses.

### Patients and tissue specimens

A total number of 73 fresh HNSCC samples and paired adjacent non-tumor epithelial tissues was obtained from patients between Jan. 2007 to Dec. 2015 at Department of oral and maxillofacial surgery, Affiliated Hospital of Stomatology, Nanjing Medical University. All samples were harvested within 30 min after surgical resection and histopathologically confirmed by senior pathologists. Seventy-six primary archived HSNCC samples from another independent patient cohort with detailed follow-up data were used for immunohistochemical staining. Patient inclusion criteria for these two cohorts were described as follows: primary HNSCC without any prior history of chemotherapy or radiotherapy; patients underwent radical tumor resection and elective or therapeutic neck dissection as required; detailed demographic, clinical and pathological data. Written informed consent was obtained from these patients or donors. This study protocol was reviewed and approved by the Research Ethic Committee of Nanjing Medical University.

### Immunohistochemical staining and scoring

Immunohistochemical staining was routinely performed on 4μm-thick sections from formalin-fixed paraffin-embedded clinical samples as previously described. Negative controls (without primary antibody incubation) were included in each staining run. Immunoreactivity was semi-quantitatively evaluated according to staining intensity and distribution using the immunoreactive score which was calculated as intensity score × proportion score as we previously reported^23,43,44^. Intensity score was defined as 0, negative; 1, weak; 2, moderate; 3, strong. The proportion score was defined as 0, negative; 1, < 10%; 2, 11–50%; 3, 51–80%; 4, > 80% positive cells. The total score ranged from 0 to 12. Accordingly, the immunoreactivity of each slide was categorized into three subgroups based on final scores: 0, negative; 1–4, low expression; 4–12, high expression.

### Bioinformatics analyses of online transcriptional profiling data of HNSCC

The original data concerning the expression of TAZ and SOX2 in HNSCC were retrieved from publicly available databases The Cancer Genome Atlas (TCGA, https://cancergenome.nih.gov/) and Gene Expression Omnibus (GEO) Database (GSE23036 and GSE65858)^45,46^. The mRNA expression levels of TAZ and SOX2 HNSCC were log2-transformed and compared using the Pearson correlation test.

### Identification of TAZ/SOX2-correlated gene signature with prognostic value

The positively or negatively correlated genes of TAZ and SOX2 in HNSCC were initially downloaded from cBioPortal (https://www.cbioportal.org/) using TCGA-HNSCC dataset. The overlapped genes were considered as potential candidates for TAZ/SOX2-correlated gene signature, which were subjected to univariate regression assay to identify candidates which were significantly associated with overall survival (with *P* value of less than 0.05) in TCGA-HNSCC datasets. Then, these candidates were further filtered using Robust likelihood-based modeling for 1000 times via R environment with Rbsurv package and multivariate Cox regression analysis with top statistical significance^44,47,48^. A risk score formula based on the expression level and coefficient of these selected candidates was generated and its optimal cut-off point was selected at the maximal sensitivity and specificity by receiver operating characteristics (ROC) curve. Subsequently, the prognostic values of this score were validated in two publically available HNSCC cohorts (GSE41613 and GSE42743)^49^.

### Statistical analysis

All quantitative data in the present study were shown as mean ± SD of three independent experiments unless otherwise stated. All data were analyzed using GraphPad Prism 7.0 or SPSS 22.0 software with Student’s *t*-test and ANOVA with post-hoc test unless otherwise specified. The Chi-squared test was applied for TAZ and SOX2 expression and various clinicopathological parameters. The Kaplan–Meier method and Log-rank test were used for the assessment of patient survival. Differences were considered statistically significant at *P* < 0.05 (*) and *P* *<* 0.01 (**).

## Supplementary information

Supplementary Tables

Supplementary Figure legends

Supplementary Fig S1

Supplementary Fig S2

Supplementary Fig S3

Supplementary Fig S4

Supplementary Fig S5

Conflict of interest statement
