# Correction: The Hippo effector TAZ promotes cancer stemness by transcriptional activation of SOX2 in head neck squamous cell carcinoma

**DOI:** 10.1038/s41419-024-07280-7

**Published:** 2024-12-16

**Authors:** Jin Li, Zhongwu Li, Yaping Wu, Yanling Wang, Dongmiao Wang, Wei Zhang, Hua Yuan, Jinhai Ye, Xiaomeng Song, Jianrong Yang, Hongbing Jiang, Jie Cheng

**Affiliations:** 1 Jiangsu Key Laboratory of Oral Disease, Nanjing Medical University, Nanjing, 210029 PR China; 2 Department of Oral and Maxillofacial Surgery, Affiliated Stomatological Hospital, Nanjing Medical University, Nanjing, 210029 PR China; 3 Department of Oral Pathology, Affiliated Stomatological Hospital, Nanjing Medical University, Nanjing, 210029 PR China

Correction to: *Cell Death* and *Disease* 10.1038/s41419-019-1838-0, published online 09 August 2019

During reading this paper, the authors noticed some unintentional errs and then retrieved the original datasets and confirmed that these errs were inadvertent during figure/manuscript preparation.

1. The GAPDH blotting images for Fadu cells in both Fig. 1a (right panel) and Fig. 3a were originated from a single-round WB experiment using the same cell lysates, which appeared to be duplicated.
2. The protein blotting images for Cal27/Fadu cell subpopulations in Fig. 2f were mistakenly assembled during figure preparation.
3. The protein blotting images for Phospho-TAZ (Ser89) in Supplementary Fig. 1c and 1e were inadvertently duplicated during figure preparation.

Although these mistakes do not affect the conclusions of this study, the authors sincerely apologize for any inconvenience or confusion this may have caused. They believe that this correction is necessary and appropriate.

**Corrected Figure 2** 
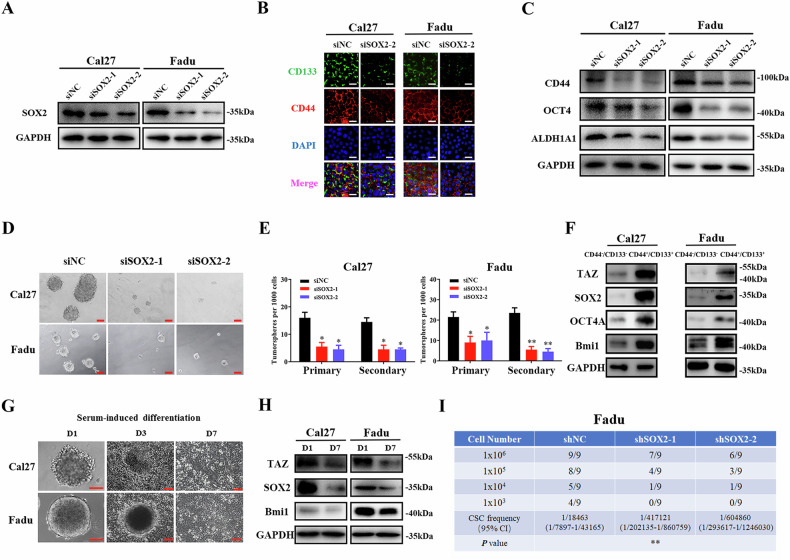


**Original Figure 2** 
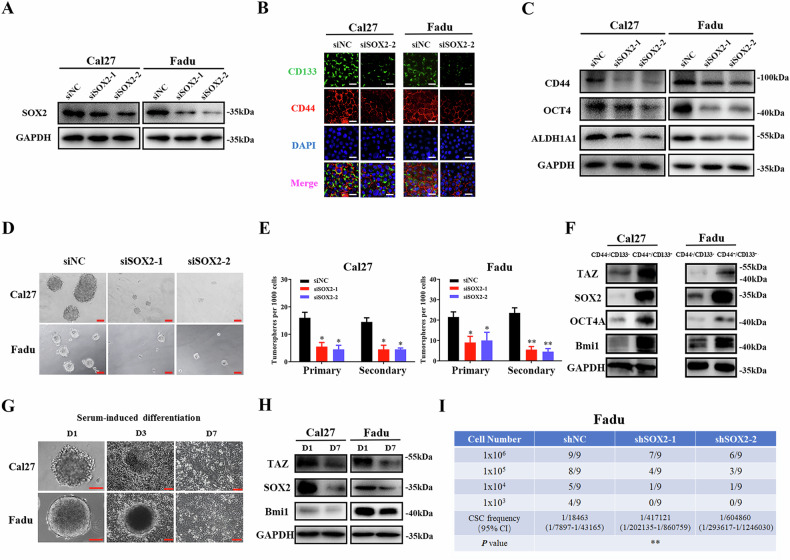


## Supplementary information

Original Supplementary Fig S1

Corrected Supplementary Fig S1

