# Supplementary material for: The Hippo effector TAZ promotes cancer stemness by transcriptional activation of SOX2 in head neck squamous cell carcinoma
Source: Cell Death Dis. 2019 Aug 9;10(8):603. doi: 10.1038/s41419-019-1838-0 (PMC6689034; doi:10.1038/s41419-019-1838-0)
Supplement: Supplementary file 1 — Supplementary Tables [file 41419_2019_1838_MOESM1_ESM.docx]

**Supplementary Table S1.** **shRNA and siRNA sequences**

| **RNAi** | **Target** | **Sequence** |
| --- | --- | --- |
| shTAZ-1 | Human TAZ | AGGTACTTCCTCAATCACA |
| shTAZ-2 | Human TAZ | ACGTTGACTTAGGAACTTT |
| shNC-1 |  | TTCTCCGAACGTGTCACGT |
| siSOX2-1/shSOX2-1  siSOX2-2/shSOX2-2  siNC-1  siTEAD4-1  siTEAD4-2  siNC-2 | Human SOX2-1  Human SOX2-2  Human TEAD 4-1  Human TEAD 4-2 | GCUCGCAGACCUACAUGAATT  ACAUGAAGAAGGAUAAGUA  TTCTCCGAACGTGTCACGT  CCGCCAAAUCUAUGACAAATT  CGCUCUGUGAGUACAUGAUTT  UUCUCCGAACGUGUCACGUTT |

**Supplementary Table S2.** **qPCR primer sequences**

| **Target (human)** | **Forward (5’ to 3’)** | **Reverse (5’ to 3’)** |
| --- | --- | --- |
| **TAZ** | GGCTGGGAGATGACCTTCAC | AGGCACTGGTGTGGAACTGAC |
| **CTGF** | GCCACAAGCTGTCCAGTCTAATCG | TGCATTCTCCAGCCATCAAGAGAC |
| **Cyr61** | ATGAATTGATTGCAGTTGGAAA | TAAAGGGTTGTATAGGATGCGA |
| **SOX2** | CCCACCTACAGCATGTCCTACTC | TGGAGTGGGAGGAAGAGGTAAC |
| **CD44** | CGGACACCATGGACAAGTTT | GAAAGCCTTGCAGAGGTCAG |
| **CD133** | GGACCCATTGGCATTCTC | CAGGACACAGCATAGAATAATC |
| **Nanog** | TTTGTGGGCCTGAAGAAAACT | AGGGCTGTCCTGAATAAGCAG |
| **OCT4A** | GTGTTCAGCCAAAAGACCATCT | GGCCTGCATGAGGGTTTCT |
| **ALDH1A1** | CCGTGGCGTACTATGGATGC | GCAGCAGACGATCTCTTTCGAT |
| **18S rRNA** | ACACGGACAGGATTGACAGA | GGACATCTAAGGGCATCACA |
| **GAPDH** | AGGTGAAGGTCGGAGTCAAC | AGTTGAGGTCAATGAAGGGG |

**SupplementaryTable S3.** **Antibodies used**

| **Specificity** | **Source** | **Catalog number** | **Application (Concentration)** |
| --- | --- | --- | --- |
| TAZ | CST | #70148 | IP（1:50）；IF (1:200); WB (1:1000) |
| TAZ | Proteintech | 66500-1-Ig | IHC (1:300); WB (1:500) |
| Phospho-TAZ (Ser89) | CST | #59971 | WB (1:1000) |
| Flag | Sigma | F3615 | WB (1:3000) |
| Bmi1 | CST | #6964 | WB (1:1000) |
| SOX2 | CST | #3579 | IF (1:200); IHC (1:200); WB (1:1000) |
| TEAD4 | Abcam | ab58310 | WB (1:500) |
| CTGF | Santa Cruz | sc-14939 | WB (1:500) |
| ALDH1A1 | Proteintech | 15910-1-AP | IF (1:200); IHC（1:150）;WB (1:2000) |
| Ki67 | Dako | M724029-2 | IHC (1:250) |
| CD44 | CST | #3570 | IF (1:200); IHC（1:200） |
| CD44  CD133 | Proteintech  CST | 60224-1-Ig  #64326 | WB (1:1000)  IF (1:200); IHC（1:200） |
| CD133  Nanog | Proteintech  CST | 18470-1-AP  #4903 | WB (1:1000)  WB (1:1000) |
| OCT4 | CST | #2890 | WB (1:1000) |
| GAPDH | CST | #2118 | WB (1:2000) |

**Supplementary Table S4.** **ChIP primer sequences**

| **Target (human)** | **Forward (5’ to 3’)** | **Reverse (5’ to 3’)** |
| --- | --- | --- |
| **CTGF** | TAGGTACCAGTGTGCCAGCTTTTTCAGAC | CATCTCGAGCGAGCTGGAGGGTGGAGT |
| **SOX2 Binding Site 1 (-293~-495)** | AGAGCTGAGTTGGACAGGGA | CTCTCACGCCCTTCTCACAG |
| **SOX2 Binding Site 2 (-1205~--1300)** | AGTTTGAGCCCCAGGCTTAAG | GAGAGAAATACCCTTACTCA |

**Supplementary Table S5.** **High TAZ/SOX2 expression and their associations with clinicopathological parameters in 76 patients with HNSCC**

| **Parameters** |  | **TAZ** | | ***P*-values** | **SOX2** | | ***P*-values** |
| --- | --- | --- | --- | --- | --- | --- | --- |
|  |  | **Low*** | **High** |  | **Low** | **High** |  |
| **Gender** | 76 | 40 | 36 |  | 46 | 30 |  |
| Male | 43 | 22 | 21 | 0.76 | 24 | 19 | 0.33 |
| Female | 33 | 18 | 15 |  | 22 | 11 |  |
| **Age** |  |  |  |  |  |  |  |
| <60 | 32 | 20 | 12 | 0.14 | 20 | 12 | 0.21 |
| ≥60 | 44 | 20 | 24 |  | 16 | 18 |  |
| **Smoking** |  |  |  |  |  |  |  |
| No | 45 | 22 | 23 | 0.43 | 31 | 14 | 0.07 |
| Yes | 31 | 18 | 13 |  | 15 | 16 |  |
| **Alcohol use** |  |  |  |  |  |  |  |
| No | 42 | 24 | 18 | 0.38 | 24 | 18 | 0.50 |
| Yes | 34 | 16 | 18 |  | 22 | 12 |  |
| **Tumor size** |  |  |  |  |  |  |  |
| T1-T2 | 45 | 29 | 16 | **0.01** | 30 | 15 | 0.18 |
| T3-T4 | 31 | 11 | 20 |  | 16 | 15 |  |
| **Pathological grade** |  |  |  |  |  |  |  |
| Ⅰ | 42 | 20 | 22 | 0.33 | 24 | 18 | 0.50 |
| Ⅱ-Ⅲ | 34 | 20 | 14 |  | 22 | 12 |  |
| **Cervical node metastasis** |  |  |  |  |  |  |  |
| N(0) | 44 | 28 | 16 | **0.02** | 34 | 10 | **0.00** |
| N(+) | 32 | 12 | 20 |  | 12 | 20 |  |
| **Clinical stage** |  |  |  |  |  |  |  |
| Ⅰ-Ⅱ | 40 | 22 | 18 | 0.66 | 24 | 16 | 0.09 |
| Ⅲ-Ⅳ | 36 | 18 | 18 |  | 22 | 14 |  |

**Supplementary Table S6.** **Univariate and multivariate Cox regression analyses of risk score in TCGA-HNSCC dataset**

|  | | | | | | | | | | | |
| --- | --- | --- | --- | --- | --- | --- | --- | --- | --- | --- | --- |
| **Variables** | | **Univariate analyses** | | | |  | | **Multivariate analyses** | | | |
|  |  | **HR [95% CI]** | | ***P*** | |  | | **HR [95% CI]** | | ***P*** | |
| **Combined cohort** | |  | |  | |  | |  | |  | |
| Age (≥60, <60) | | 1.291(0.981-1.700) | | 0.069 | |  | |  | |  | |
| Gender (male, female) | | 0.754(0.566-1.004) | | 0.054 | |  | |  | |  | |
| Smoking history category (≥3, <3) | | 0.802(0.606-1.062) | | 0.123 | |  | |  | |  | |
| Alcohol use (Yes, No) | | 0.978(0.734-1.304) | | 0.880 | |  | |  | |  | |
| Tumor size (T3-T4, T1-T2) | | 1.569(1.143-2.153) | | **0.005** | |  | | 1.627(0.924-2.866) | | 0.092 | |
| Pathological grade (III-IV, I-II) | | 1.743(1.177-2.580) | | **0.006** | |  | | 1.197(0.665-2.156) | | 0.548 | |
| Cervical node metastasis (N+, N0) | | 1.383(1.039-1.842) | | **0.026** | |  | | 1.323(0.913-1.917) | | 0.140 | |
| Clinical stage (III-IV, I-II) | | 1.257(0.901-1.753) | | 0.178 | |  | | 0.712(0.414-1.223) | | 0.218 | |
| Risk score (High, Low) | | 2.387(1.824-3.124) | | **<0.001** | |  | | 2.107(1.551-2.863) | | **<0.001** | |
| HR, hazard ratio; CI, confidence interval. | |  | |  | |  | |  | |  | |
