# Supplementary material for: The Hippo effector TAZ promotes cancer stemness by transcriptional activation of SOX2 in head neck squamous cell carcinoma
Source: Cell Death Dis. 2019 Aug 9;10(8):603. doi: 10.1038/s41419-019-1838-0 (PMC6689034; doi:10.1038/s41419-019-1838-0)
Supplement: Supplementary file 2 — Supplementary Figure legends [file 41419_2019_1838_MOESM2_ESM.docx]

**Supplementary Figure S1**

**a-f:** The mRNA and protein expression changes of TAZ and its downstream target CYR61 were measured by qRT-PCR and western blot in HEK293T (**a, b**), Fadu (**c, d**) and HN6 (**e, f**) cells infected with SOX2 overexpressing lentivirus. Data were presented as mean ± SD from three independent experiments. **P* < 0.05, ***P* < 0.01, ns *P*> 0.05.

**Supplementary Figure S2**

**a:** CD44^+^CD133^+^ and CD44^-^CD133^-^ subpopulations were isolated from Cal27 cells by FACS.

**b, c:** Representative images of tumorsphere formed from CD44^+^CD133^+^ and CD44^-^CD133^-^ subpopulations were shown. Scale bar: 100μm.

**d:** Immunofluorescence staining of CD44 and CD133 in tumorsphere was shown. DAPI was used for DNA staining. Scale bar: 50μm.

**e, f:** In vivo tumor-initiating frequencies of CD44^+^CD133^+^ and CD44^-^CD133^-^ subpopulations isolated from Cal27 (**e**) and Fadu (**f**) cells were calculated using ELDA software (http://bioinf.wehi.edu.au/software/elda/).

**Supplementary Figure S3**

**a, b:** Cell proliferation was measured by CCK-8 viability assay (**A**) and BrdU incorporation assay (**B**) in Cal27 and Fadu cells after SOX2 knockdown. BrdU^+^ and DAPI^+^ cells were identified in five representative fields from each condition and then counted via ImageJ software. Representative images were shown. Data were presented as mean ± SD from 3 independent experiments. Scale bar: 100μm.

**c, d:** Percentages of apoptotic cells were determined by Annexin V-PI double staining in Cal27 and Fadu cells after SOX2 knockdown. Data were presented as Mean ± SD from three independent experiments, **P* < 0.05, ***P* < 0.01.

**Supplementary Figure S4**

**a-c:** Significant correlation between TAZ and SOX2 mRNA in primary HNSCC from public databases (a: TCGA, n=502; b: GSE23036 n=63; C: GSE65858 n=270) was found. Sample correlation coefficient (r) was calculated using the linear regression analysis (F-test, *P*< 0.0001).

**Supplementary Figure S5**

**a, d:** Distribution of TAZ/SOX2-correlated gene signature and expression of each mRNA in GSE41613 (testing) and GSE42743 (validation) datasets was shown.

**b, e:** The sensitivity and specificity of TAZ/SOX2-correlated gene signature in prognostic prediction were confirmed in GSE41613 (testing) and GSE42743 (validation) cohorts.

**c, f:** High or low TAZ/SOX2-correlated gene signature robustly stratified patients into subgroups with low or high survival ratios (Log-rank test).
