# Supplementary figures and images for: The Hippo effector TAZ promotes cancer stemness by transcriptional activation of SOX2 in head neck squamous cell carcinoma
Source: Cell Death Dis. 2019 Aug 9;10(8):603. doi: 10.1038/s41419-019-1838-0 (PMC6689034; doi:10.1038/s41419-019-1838-0)

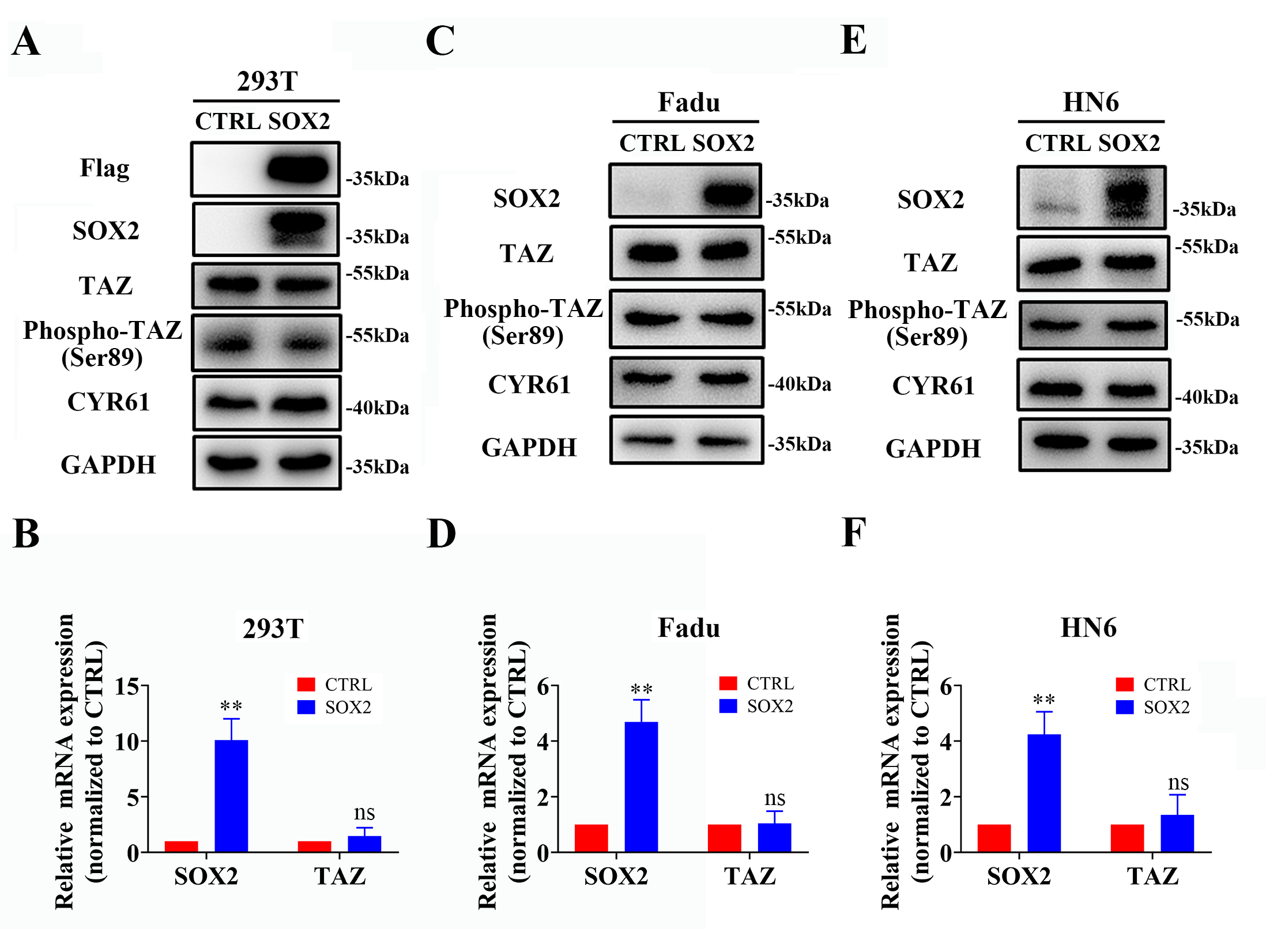

Supplement: Supplementary file 3 — Supplementary Fig S1 [file 41419_2019_1838_MOESM3_ESM.tif]

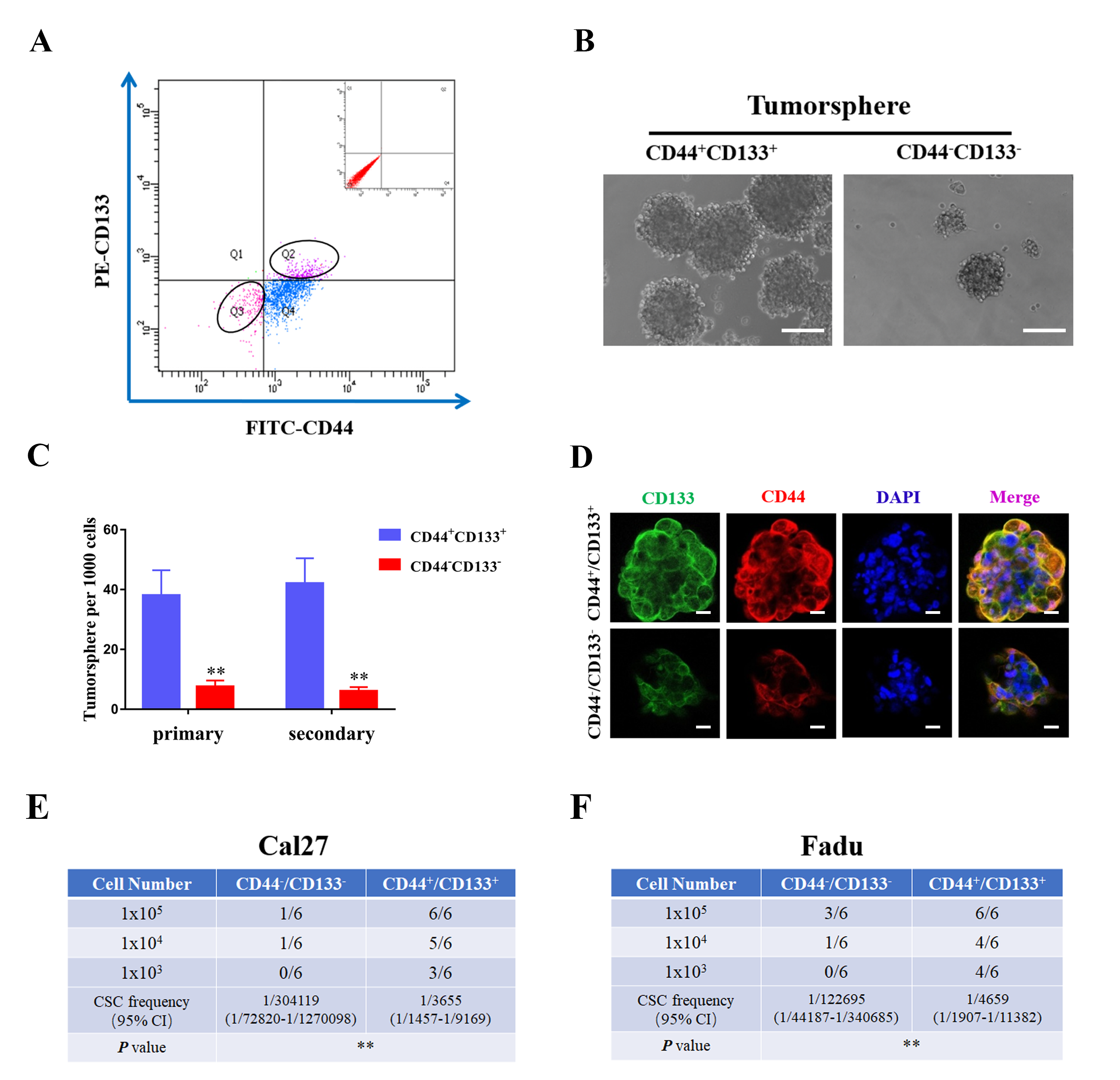

Supplement: Supplementary file 4 — Supplementary Fig S2 [file 41419_2019_1838_MOESM4_ESM.tif]

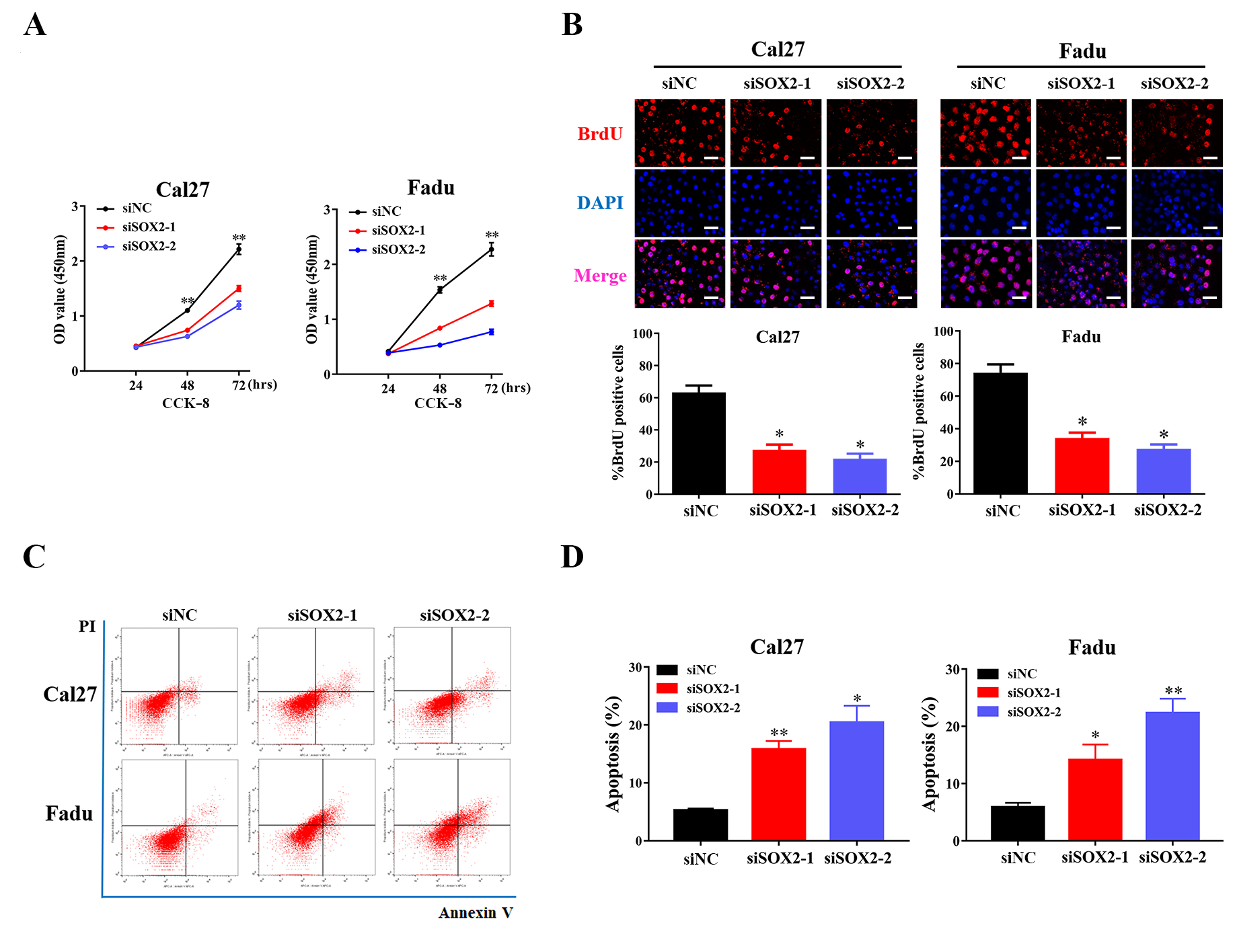

Supplement: Supplementary file 5 — Supplementary Fig S3 [file 41419_2019_1838_MOESM5_ESM.tif]

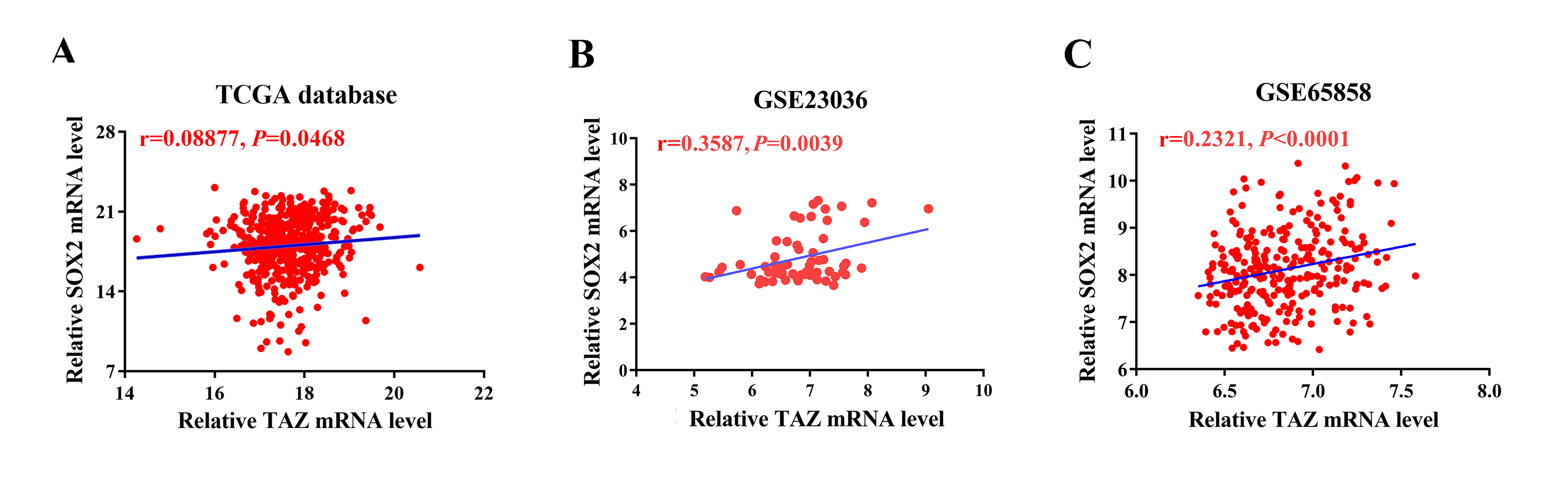

Supplement: Supplementary file 6 — Supplementary Fig S4 [file 41419_2019_1838_MOESM6_ESM.tif]

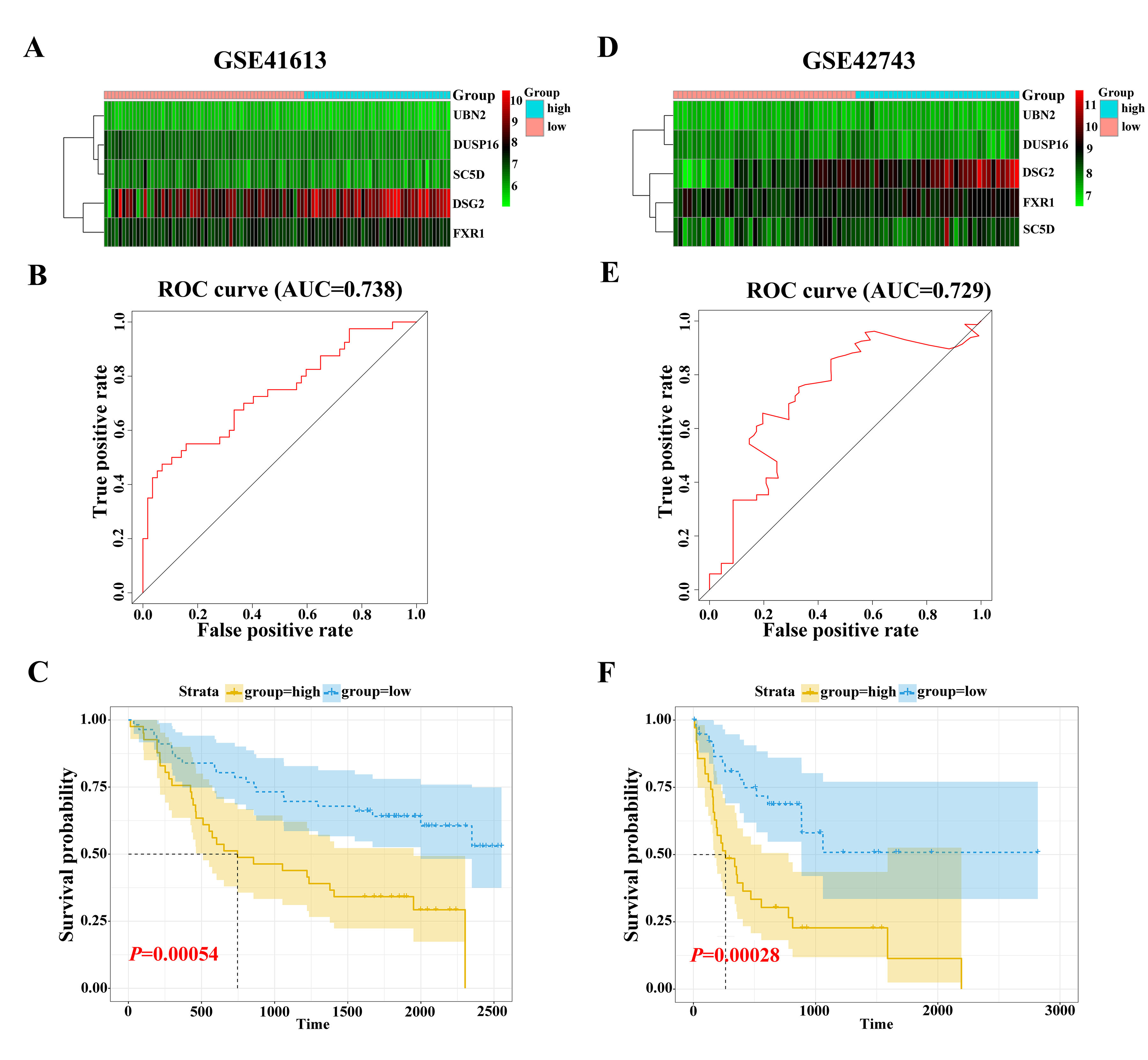

Supplement: Supplementary file 7 — Supplementary Fig S5 [file 41419_2019_1838_MOESM7_ESM.tif]
