# Supplementary material for: The Hippo effector TAZ promotes cancer stemness by transcriptional activation of SOX2 in head neck squamous cell carcinoma
Source: Cell Death Dis. 2019 Aug 9;10(8):603. doi: 10.1038/s41419-019-1838-0 (PMC6689034; doi:10.1038/s41419-019-1838-0)
Supplement: Supplementary file 8 — Conflict of interest statement [file 41419_2019_1838_MOESM8_ESM.docx]

**Conflict of Interest Statement**

The authors declared that they had no conflicts of interests to this work.
