# Supplementary figures and images for: Correction: The Hippo effector TAZ promotes cancer stemness by transcriptional activation of SOX2 in head neck squamous cell carcinoma
Source: Cell Death Dis. 2024 Dec 16;15(12):887. doi: 10.1038/s41419-024-07280-7 (PMC11649931; doi:10.1038/s41419-024-07280-7)

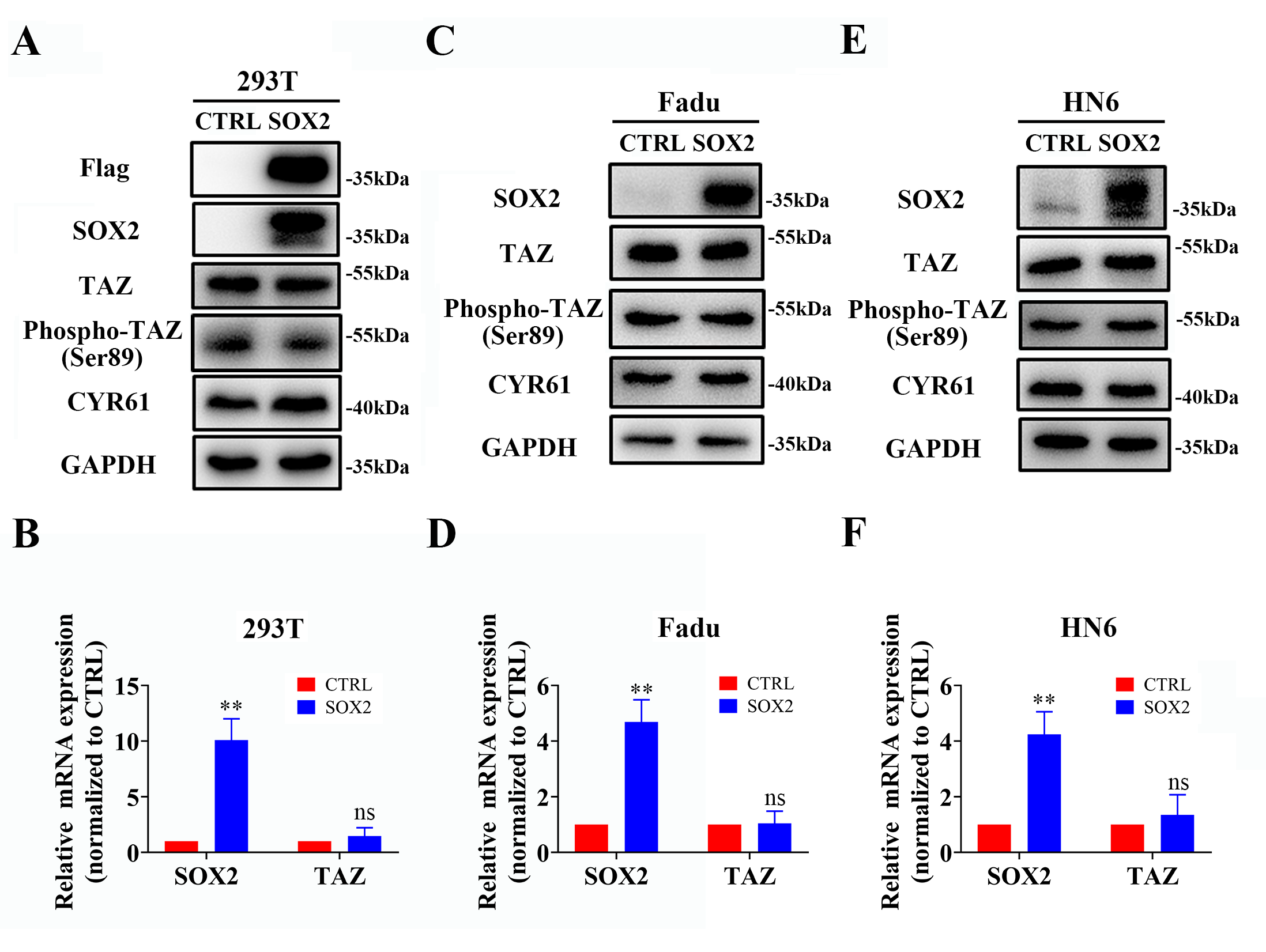

Supplement: Supplementary file 1 — Original Supplementary Fig S1 [file 41419_2024_7280_MOESM1_ESM.tiff]

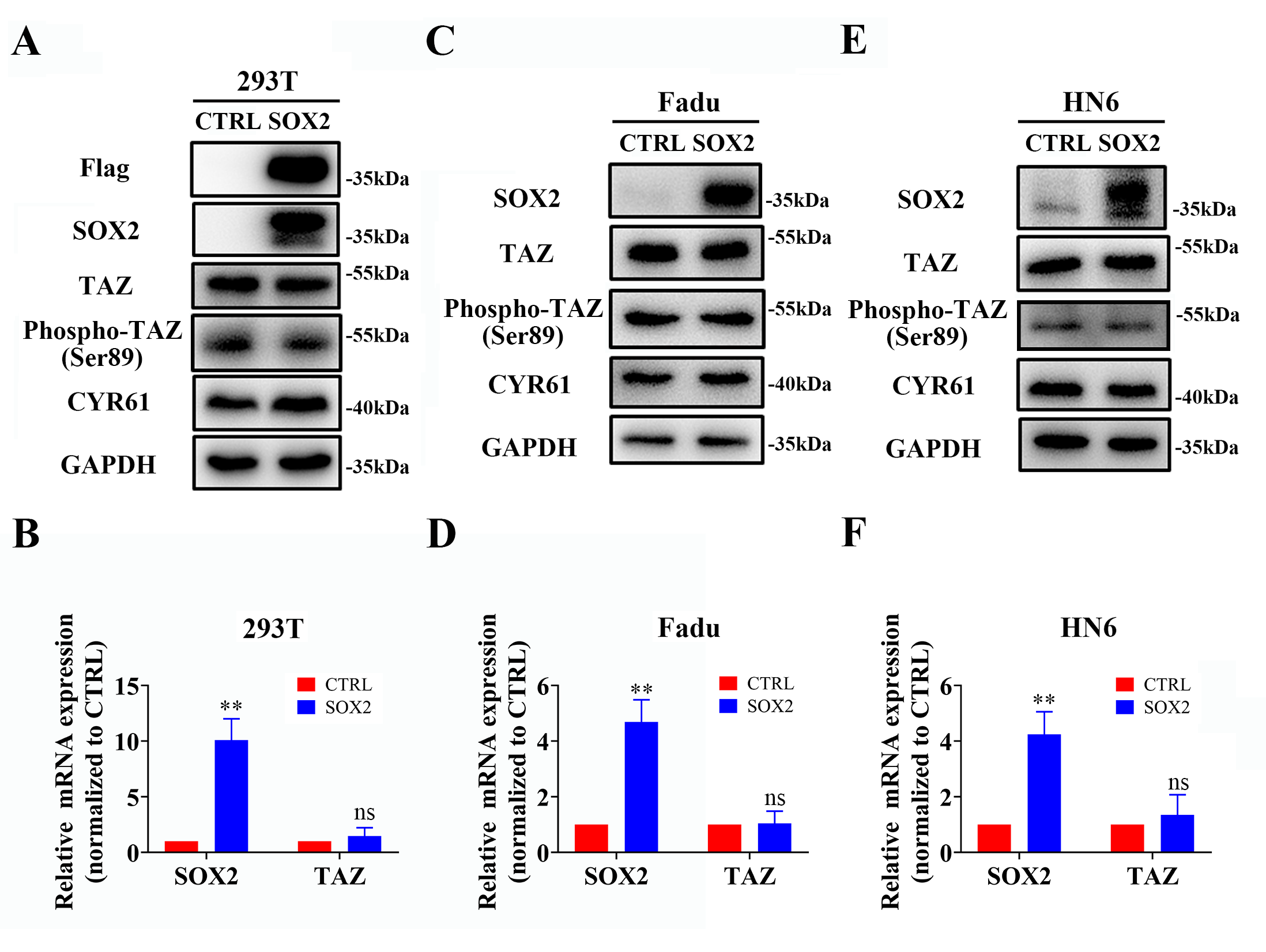

Supplement: Supplementary file 2 — Corrected Supplementary Fig S1 [file 41419_2024_7280_MOESM2_ESM.tiff]
